# Remote Sensing of CDOM, CDOM Spectral Slope, and Dissolved Organic Carbon in the Global Ocean

**DOI:** 10.3390/app8122687

**Published:** 2018-12-19

**Authors:** Dirk Aurin, Antonio Mannino, David J. Lary

**Affiliations:** 1 NASA Goddard Space Flight Center (USRA), Greenbelt, MD 20771, USA; 2 NASA Goddard Space Flight Center, Greenbelt, MD 20771, USA; antonio.mannino-1@nasa.gov; 3 University of Texas at Dallas, Richardson, TX 75080, USA; david.lary@utdallas.edu

**Keywords:** ocean color database, oceanic carbon, chromophoric dissolved organic matter, dissolved organic carbon, CDOM spectral slope, ocean color remote sensing, algorithm development, ocean color algorithm validation, ocean optics, CDOM climatology, CDOM and ENSO, machine learning

## Abstract

A Global Ocean Carbon Algorithm Database (GOCAD) has been developed from over 500 oceanographic field campaigns conducted worldwide over the past 30 years including in situ reflectances and coincident satellite imagery, multi- and hyperspectral Chromophoric Dissolved Organic Matter (CDOM) absorption coefficients from 245–715 nm, CDOM spectral slopes in eight visible and ultraviolet wavebands, dissolved and particulate organic carbon (DOC and POC, respectively), and inherent optical, physical, and biogeochemical properties. From field optical and radiometric data and satellite measurements, several semi-analytical, empirical, and machine learning algorithms for retrieving global DOC, CDOM, and CDOM slope were developed, optimized for global retrieval, and validated. Global climatologies of satellite-retrieved CDOM absorption coefficient and spectral slope based on the most robust of these algorithms lag seasonal patterns of phytoplankton biomass belying Case 1 assumptions, and track terrestrial runoff on ocean basin scales. Variability in satellite retrievals of CDOM absorption and spectral slope anomalies are tightly coupled to changes in atmospheric and oceanographic conditions associated with El Niño Southern Oscillation (ENSO), strongly covary with the multivariate ENSO index in a large region of the tropical Pacific, and provide insights into the potential evolution and feedbacks related to sea surface dissolved carbon in a warming climate. Further validation of the DOC algorithm developed here is warranted to better characterize its limitations, particularly in mid-ocean gyres and the southern oceans.

## 1. Introduction

### 1.1. Background

In 1896, Svante Arrhenius introduced the theory that adding carbon dioxide (CO_2_) to the atmosphere enhances the planetary greenhouse effect. Over the intervening century, it became clear that the marine dissolved organic carbon (DOC) pool comprised the vast majority of the organic carbon in the oceans, and was nearly equivalent to the atmospheric pool of CO_2_ [1]. In fact, remineralization of just 1% of the DOC in the oceans (e.g., by microbial metabolism and photo-oxidation) would generate a flux of CO_2_ into the atmosphere greater than that resulting from all the fossil fuel burned in a year [2]. Recently, Belanger et al. [3] estimated that photoproduction of CO_2_ from Chromophoric Dissolved Organic Matter (CDOM) has already increased by ~15% in Arctic waters due to an increase in ultraviolet radiation and the decrease in sea ice associated with global warming. Positive feedbacks such as this have potentially serious consequences for humans and ecosystems alike, and emphasize the urgency to develop robust, global algorithms for retrieving oceanic carbon products remotely and synoptically.

CDOM (refer to Table 1 for terms and abbreviations) is used to describe an often difficult to define fraction of the DOC pool (see Section 1.4) which has historically been called gilvin, *gelbstoff*, or simply “yellow substance”. As its name suggests, the presence of CDOM imparts color to the water column through absorption of light by various chromophores, thereby providing an effective means of detecting CDOM remotely from ocean color reflection. Found in all natural waters and generally in highest concentration near shore, CDOM results from the breakdown products of plants and other organic matter into humic materials, and plays a significant role in aquatic photochemistry, photobiology, and as a tracer of the origins of oceanic water masses, e.g., [4,5]. DOC and CDOM can be terrigenous or autochthonous (i.e., deriving from in situ primary and bacterial production in river to ocean waters), with the DOC variously composed of high molecular weight (HMW) humic substances (which tend to be more labile) and low molecular weight (LMW) humics (such as fulvic acids), depending on its origin, labile fraction, age, and whether it has transitioned from fresh waters to marine [6–12]. Most estuarine and nearshore CDOM is terrigenous, and as it mixes in rivers on its transit to marine waters, the amount of HMW material declines from flocculation, photo-oxidation and microbial decomposition leaving marine waters dominated by LMW CDOM (e.g., [6]), a condition imparting a characteristic spectral shape to inherent light absorption by CDOM (*a_g_*(λ), where λ is wavelength) [7]. Inherent optical properties (IOPs) of the water column, such as the absorption and backscattering coefficients, depend on the composition and concentration of the dissolved and suspended material present, as well as the size and structure of the particles, and water itself. CDOM concentration—for which *a_g_*(λ) is the common proxy following Beer’s law—varies widely in the ocean, tending to be highest near river outflows, but may also be high in upwelling regions and other regions of autochthonous, plankton-based production through exudation, excretion, and microbial breakdown of detritus [8]. It is degraded over time both by microbial activity, photooxidation, and other abiotic processes, ultimately resulting in remineralization of the carbon, and release from the ocean as CO and CO_2_. In the case of the CDOM fraction of DOC, degradation over time scales of days to millennia can significantly change the magnitude and spectral characteristics of *a_g_*(λ).

CDOM absorption is a superposition of the spectral absorption by its varied chromophores, and increases roughly exponentially (or hyperbolically [9]) with decreasing wavelength in the visible (VIS) and ultraviolet (UV) spectral ranges, as described in the next section. CDOM tends to dominate the blue and UV spectrum in many coastal and estuarine environments (e.g., [7,10–12]), and is the most important factor controlling UV and blue light penetration even in the open ocean [13] despite its generally lower concentration and distance from land. Within the visible spectrum, *a_g_*(λ) reduces the photosynthetically available radiation supporting phytoplankton and macrophytic growth, and generates heat in the surface layer of the water column, thus affecting mixing [14]. In the UV, CDOM causes surface heating as well, but also acts to protectively shade aquatic organisms, thus reducing the amount of damaging high frequency radiation reaching vulnerable cell structures.

From the passive remote sensing perspective, CDOM reduces the amount of blue light available for reflection out of the water column, and can therefore have a significant impact on ocean color algorithms, for example increasing uncertainty in blue-green band-ratio algorithms designed to estimate chlorophyll-*a* concentration (Chl) from its absorption peak near 443 nm [13,15]. These types of Chl algorithms assume covariance in Chl, CDOM, and other water column constituents (i.e., the “Case 1 waters” assumption [16]). By contrast, semi-analytical algorithms (SAAs) that invert the ocean color signal to retrieve individual component absorption spectra (particles, CDOM, water) are stymied by the presence of non-algal particulates (NAPs), which have a similar spectral shape to CDOM. As a result, these approaches tend to retrieve only the sum of these two elements [17] (and references therein).

### 1.2. Spectral Shape of CDOM

The CDOM absorption coefficient is generally modeled with an exponentially decaying function with increasing wavelength, λ.

(1) $$a_{g}(\lambda )=a_{g}(\lambda_{0})\;e^{-S_{g}(\lambda -\lambda_{0})}$$

where *S_g_* is the spectral slope parameter and λ_0_ is a reference wavelength. *S_g_* in various spectral ranges in the UV and VIS contains information about CDOM’s photoreactive state, chemical composition, molecular weight distribution, and origin [4,7,18–21].

While the single exponential model in Equation (1) is accurate within limited wavebands, CDOM spectral slope, *S_g_*, is not constant across the UV and VIS and depends on the wavelength range used, spectral resolution, and reference wavelength *λ*_0_. Furthermore, comparative analysis of CDOM spectral shape as reported in the literature has been confounded by the multitude of methodologies and reference wavebands used historically to calculate *S_g_* [9]. For instance, a linear fit to logarithmically transformed *a_g_* data yields results for *S_g_* biased by higher wavelength absorption, whereas a least-squared difference minimization fitting favors the lower wavelengths where the magnitude of *a_g_* is higher, and is considered more accurate [7,22]. Changes to *S_g_* resulting from photodegradation are wavelength dependent, i.e., increasing below 460 nm and decreasing above 510 nm [23], although when calculated across the VIS from 412–555, slope is expected to increase through the destruction of large humic complexes resulting in lower molecular weight CDOM [24]. This effect appears to reverse over time as more refractory, low-molecular weight compounds are also degraded, thereby reducing CDOM absorption at shorter wavelengths relative to longer, and decreasing spectral slope across the VIS.

All these factors lead to challenges in comparing CDOM spectral slope between studies, and a more standardized approach to CDOM spectral shape measurement still seems warranted [19]. The concept of the spectral slope curve, *S_g_*(*λ*)—analogous to the first derivative of Sg with respect to λ—was explored by Loiselle et al. in 2009 [23]. Calculating *S_g_* from natural waters, cultures, and laboratory standards at 20 nm waveband intervals between 200–700 nm, they showed that *S_g_*(λ) had complex spectral characteristics including peaks near 390 nm likely indicating a prevalence of autochthonous production of fulvic acid-type CDOM, and near 280 nm possibly due to the release of proteins or phenols by phytoplankton. While the spectral slope curve approach of Loiselle et al. 2009 represents an elegant method for quantifying many subtle characteristics of CDOM spectral shape when compared to, for example, using a single slope parameter across the UV and VIS, it does require relatively high spectral resolution data collection. Historically, this was not always available or reported, and here we focus on a set of eight different spectral ranges commonly seen in the literature and described in detail below.

### 1.3. Remotely Sensing CDOM and S_g_

As interest in CDOM has grown in recent years, numerous empirical ocean color algorithms for retrieving CDOM within limited geographic regions have emerged, e.g., [25–30]. A smaller number of more generally applicable, global empirical algorithms have also been developed, including one for retrieving a unitless index of CDOM prevalence, though it does not retrieve *a_g_*(λ) or *S_g_* and depends upon Case 1 assumptions. More recently, Tiwari and Shanmugam published global empirical algorithms for both *a_g_*(λ) and *S_g_* [31,32]. These were optimized and tested using field data aggregated in NOMAD (the NASA bio-Optical Marine Algorithm Dataset version 2 [33]) and the synthetic ocean color dataset developed by the International Ocean Colour Coordinating Group (IOCCG) for the purpose of algorithm development and validation [34].

Other approaches to retrieving CDOM remotely depend on the premise that sea-surface reflectance is approximately inversely proportional to the total absorption coefficient [16,35,36], which can be linearly separated into various contributions by particulate and dissolved constituents. This forms the basis to semi-analytical ocean color algorithms (SAAs) for retrieving constituent absorption, e.g., [37–39], but, as already mentioned, owing to the similarity in spectral shape of non-algal particulate (i.e., detrital, microbial, and sedimentary) absorption and *a_g_*(λ), SAAs generally retrieve only their sum, *a_dg_* [17]. To circumvent this difficulty, empirical methods are sometimes added to SAAs to help distinguish non-algal from dissolved absorption [40–45].

### 1.4. Remotely Sensing DOC

One of the most challenging aspects of developing robust, global ocean color algorithms for DOC is that the relationship between DOC and CDOM (i.e., the DOC-specific absorption) is highly variable, in some cases negatively correlated (e.g., Southern Ocean, [46]) and often poorly defined, particularly in open ocean areas such as the Sargasso Sea [47,48]. In some cases, the relationship is better constrained within a particular region and season, as shown by measurements made in the Mid-Atlantic Bight on the eastern shelf of North America [28]. Because absorption by CDOM is the only way in which ocean color is impacted by DOC, some other independent knowledge of water type is needed for retrieval of DOC from space.

### 1.5. Algorithm Development Data

One of the most confounding challenges in the development of both empirical and semi-analytical algorithms is the lack of a large, comprehensive database containing a broad enough dynamic range in optical characteristics to be representative of the majority of the world’s oceans, while also having realistic combinations of inherent optical properties, which are not guaranteed in large, synthetic, modeled datasets. NOMAD represents the first (and most recent, as of this writing) major effort to provide the ocean color community with such a dataset. It was aggregated and selected from all of the relevant field data submitted to the NASA SeaBASS archive (http://seabass.gsfc.nasa.gov/), and has been extremely useful to those in the ocean color algorithm community since its original publication in 2005 and update in 2008. However, NOMAD was not focused on CDOM. For example, while it contains about 3700 coincident radiometric and phytoplankton pigment observations, coincident radiometric and CDOM observations number just ~1200. In part, this is because CDOM data collected using in situ instrumentation were excluded for various reasons discussed at greater length below. The remaining CDOM records—those measured from discrete water samples—were modeled spectrally at the preselected NOMAD wavebands after fitting field data to Equation (1), and do not extend into the UV where spectral shape can provide useful insights into the origin and photooxidation state of CDOM. NOMAD does not contain any DOC data observations.

Using the methodology described in the next section, we extend the NOMAD approach to create a global ocean color algorithm development database better suited to DOC and its optical components, CDOM and CDOM spectral slope, ultimately including over 51,000 field observations of surface-averaged inherent optical properties. These are matched to between ~8000 and ~11,000 coincident estimates of sea surface reflectance made from in situ measurements as well as satellite imagery from SeaWiFS and MODIS Aqua and Terra instruments. The global ocean carbon algorithm database (hereafter Global Ocean Carbon Algorithm Database (GOCAD) records are split into independent sets of field stations for training/optimization (i.e., with in situ radiometry) and validation (i.e., with satellite imagery) of algorithms, as described in the Section 2. A basic overview of the most relevant aspects of the global dataset is presented in Section 3.1. In Section 3.2, empirical and SAA approaches to retrieval of DOC, CDOM, and CDOM slope are developed and discussed. Finally, algorithms are applied to global climatological satellite imagery and discussed in Section 3.3.

## 2. Methodology

### 2.1. Database Assembly Overview

Field measurements of CDOM, DOC, remote sensing reflectance, *R_rs_*(λ), and ancillary data and metadata were downloaded from SeaBASS and the Hansell/Carlson collection (https://hansell-lab.rsmas.miami.edu/research/data-collection/index.html) in April 2013. Coincident, Level 2 (L2) SeaWiFS and MODIS Aqua and Terra satellite imagery at all field stations were downloaded from the NASA Ocean Color website (http://oceancolor.gsfc.nasa.gov). Due to the size of aggregated datasets for each of the key constituents (e.g., 117,291 raw CDOM records, 31,474 raw DOC records, 115,773 in situ reflectance records, and ~177,000 matching satellite scenes), extensive automation in the processing, quality control, and merging of the databases was a necessity. A station-by-station analysis (or field experiment-specific analysis, as in [33]) of the data for establishing the customized spatial and temporal thresholds for matching coincident inherent and apparent optical properties and satellite imagery was not feasible. Relatively broad guidelines conducive to automation were established, as described in detail in the following sections. We assume, for example, that geospatial and temporal variability of CDOM and DOC is higher in coastal and shelf waters (defined here as samples collected in waters of 1000 m depth or less) than in the pelagic.

### 2.2. Field Data

Targeted searches of SeaBASS were conducted for all records containing *a_g_*, DOC, or in situ reflectances (see 2.2.3). Resultant data from the following physical, bio-optical, and biogeochemical fields were also retained where they happened to be present in SeaBASS files: depth, temperature, salinity, *a_nap_*, *a_p_*, *a_pg_*, *a_dg_*, *b_bt_*, particulate organic carbon (POC), total organic carbon (TOC), and Chl. Ancillary data including time and date, latitude, longitude, and bottom depth were also retained, as well as complete SeaBASS metadata for each record. Carbon data were downloaded from each of the data repository resources linked in the Hansell/Carlson DOM Data Collection (http://yyy.rsmas.miami.edu/groups/biogeochem/Data.html). These were also queried for all the parameters above and assigned metadata for each cruise. Table 2 provides a complete listing and overview of all the field experiments retained in the final, quality-controlled database.

#### 2.2.1. CDOM

CDOM absorption was measured in field experiments using a variety of instruments and protocols. Examples include in-line filtered (generally 0.2 μm) flow-through systems outfitted with ac-9 or ac-S absorption and attenuation meters (Wet Labs) and processed to *a_g_*(λ) [49–51], as well as discrete sampling and filtration for bench-top spectrophotometry [52], or in liquid capillary waveguides [53]. Unfortunately, SeaBASS metadata did not historically specify which methods or protocols were used in data collection or processing, but more recently (since approximately 2012), investigators have been required to submit ancillary documentation, such as instrument calibration records, and encouraged to submit documentation retroactively.

CDOM data measured in situ (i.e., with ac-9 or ac-S instruments; 33.5% of the preliminary CDOM dataset) were subject to particulate and bubble contamination, especially in experiments in which an automated in-line flow valve switched between filtered and unfiltered water presenting the opportunity for unfiltered water to reside in the plumbing during CDOM data collection. To identify and eliminate particle contamination, any CDOM records with a notable (i.e., ≥0.006 m^−1^) increase in absorption at 676 nm (a phytoplankton absorption peak) above the absorption curve from 650–715 nm were considered contaminated and removed (109 records).

Nonlinear, least squares minimization was used to fit *a_g_*(λ) to Equation (1) for calculating slopes of all hyperspectral *a_g_* into seven spectral ranges: 275–295 nm, 290–600 nm, 300–600 nm, 350–400 nm, 350–600 nm, 380–600 nm, and 412–600 nm. Multispectral ac-9 data were fitted for slope using the six wavebands in the 412–555 nm range. To reduce outliers and noisy data, any CDOM slope data found to be outside of the range 0.005–0.05 nm^−1^ were considered unrealistic and eliminated, together with the *a_g_*(λ) data used to calculate them. This accounted for only 125 hyperspectral records in the 300–600 nm range (spectrophotometric), but nearly 6,000 records in the 412–600 nm range (predominantly flow-through). To further reduce outliers and noisy records, *S_g_* and *a_g_* data were eliminated where *S_g_* in any slope range was greater than two standard deviations from the median for the entire database, or where they were outside the 2nd and 98th percentiles. This reduced the database of CDOM by nearly 11,000 records. In addition, 460 records were removed for *a_g_*(676) > 0.1 m^−1^, *a_g_*(715) > 0.05 m^−1^, or an average *a_g_*(λ > 680 nm) > 0.05 m^−1^, and an additional 1,057 CDOM records with extreme outliers (>4 standard deviations from the median) in the red (λ > 620) were eliminated.

#### 2.2.2. DOC

While DOC was included in about 850 SeaBASS records, the majority of the carbon data retained after surface and spatial binning (see 2.2.4) were from the Hansell/Carlson datasets. In total, 1957, 625, and 45 stations included DOC, POC, and TOC, respectively. Outliers (1st and 99th percentiles) were eliminated, and stations were merged with the CDOM records after surface and spatial binning. Specifically, Hansell/Carlson data were matched to CDOM field stations if they were within 1 h, 2.5 m depth, and 1 km in continental shelf waters (bottom depth ≤ 1000 m) and within 3 h, 5 m depth, and 5 km off the shelf. Multiple matches within these criteria were averaged and retained if individual measurements were with 1.5 standard deviations of the mean and the coefficient of variability of the ensemble was ≤0.25.

#### 2.2.3. In situ Reflectances

SeaBASS searches for field radiometry targeted *R_rs_* (or equivalently *L_w_* and *E_s_*, where *R_rs_* = *L_w_*/*E_s_*, or *L_wn_*, where *R_rs_* is *L_wn_* divided by the top of atmosphere solar irradiance [54]). A total of 135,966 independent field observations of *R_rs_* were binned as described in 2.2.4, quality controlled as described in 2.2.5, and then matched to the CDOM database using the same spatial, temporal, and outlier elimination criteria used for DOC (2.2.2).

#### 2.2.4. Bathymetry, Surface Averaging, and Spatial and Spectral Binning

Records with no reported bottom depth (~85% of the database) were matched to the nearest pixel in the UNESCO GEBCO 08 0.05 degree bathymetry grid (http://www.gebco.net/data_and_products/gridded_bathymetry_data/documents/gebco_08.pdf). The purpose of GOCAD is the development of satellite algorithms for surface retrievals, so data collected at depth were discarded as follows: on the continental shelf (defined here as bottom depth 1000 m or less) samples from deeper than 5 m were discarded, as were data from deeper than 10 m off the shelf (~34% of the database combined). 57,127 surface records remained. Samples collected in profile within the surface layer (top 5 m on-shelf, top 10 m off-shelf) were averaged. Samples collected in transect were additionally binned to a 0.5 km grid and averaged.

All absorption related IOPs were matched to the following wavebands with a 2.5 nm tolerance: 245 nm, 1 nm resolution between 250 and 555 nm, 560, 620, 630, 645, 650, 665, 670, 676, 680, 705, and 715 nm. Backscattering data were similarly matched to 1 nm bands from 400 to 700 nm. Hyperspectral in situ *R_rs_*(λ) were matched to both SeaWiFS bands (412, 443, 490, 510, 555, and 670 nm) and MODIS bands (412, 443 488, 531, 547, 667) by weighting the data to the instrument-specific spectral response functions for SeaWiFS, Aqua, and Terra (https://oceancolor.gsfc.nasa.gov/docs/rsr/rsr_tables/). Multispectral in situ *R_rs_*(λ) were matched to satellite bands to within a 2.5 nm tolerance.

#### 2.2.5. Additional Quality Controls

In addition to those measures already discussed for CDOM and CDOM slope outliers in Section 2.2.1, *a_d_*(λ) records were considered unrealistic and removed at all wavelengths if they exceeded 12 m^−1^ anywhere within the spectral range reported. Similarly, *a_p_*(λ) was removed if it exceeded 20 m^−1^, *b_bt_*(λ) if it exceeded 0.15 m^−1^. *R_rs_*(λ) were eliminated if they exceeded 0.075 sr^−1^ or were less than −0.001 sr^−1^ in any band, or if they were outside the 95th percentile for any given band.

### 2.3. Satellite Imagery and Matching

Ocean color satellite imagery from SeaWiFS, MODIS-Aqua, and MODIS-Terra that matched the field observations were selected and processed for further analysis. Scripted calls to the NASA GSFC Ocean Color browser (http://oceancolor.gsfc.nasa.gov/cgi/browse.pl) after the 2012.0 MODIS-Aqua reprocessing (http://oceancolor.gsfc.nasa.gov/WIKI/OCReproc20120MA.html) were used to identify and download 1 km nominal nadir resolution L2 SeaWiFS, Aqua, and Terra satellite scenes within 0.05 degrees of field observations on the same day. These were spatially extracted for a 5 × 5 pixel array around the station location. In <1% of stations, high resolution SeaWiFS imagery was not available, and Global Area Coverage (GAC; nominally 4.4 km spacing) scenes were substituted. By default, data were masked based on standard L2 flags using the criteria described in [55]: land, high solar or satellite zenith angle, clouds, sea ice, high light, stray light, glint, low water leaving radiance, and atmospheric correction failure. Extracted satellite data were then evaluated for coincidence with field sampling stations. Criteria were principally based on those of Bailey and Werdell (2006). Specifically, extracted satellite pixel arrays were retained in the database if the overpass occurred within 8 h of field sampling. For each waveband of *R_rs_*, negative and outlier pixels within each array (>1.5 standard deviations from the mean) were set to null values. Data were only retained in each waveband if greater than 50% of non-land pixels were still valid, with no fewer than five valid pixels in total. Finally, the mean *R_rs_* values for each array were calculated and retained in the database only if those pixels had a coefficient of variation (CV) < 0.25 (rather than <0.15 applied in Bailey and Werdell (2006)). Of the 50,127 field stations with spatially gridded, depth binned and quality controlled CDOM data, 8252 stations had matching quality controlled Aqua imagery, 11,156 matched Terra imagery, and 11,818 matched SeaWiFS imagery.

For the purpose of further quality assurance, several match-up metrics were retained in the final database, including the time difference, CV, the number of matched satellite pixel arrays for each *R_rs_* channel, the areal extent of the matched pixels (nominally ~25 km^2^), and the distance between the field sampling location and the central pixel (nominally < ~1 km). Sensor viewing angle, which can significantly increase error in estimates of satellite *R_rs_* due to increased uncertainties in the atmospheric correction, is not available on a pixel-by-pixel basis in standard L2 products. On the other hand, ground sample area, which can be approximated from the geographic coordinates of pixel arrays, is a good proxy for sensor viewing angle, with larger areas representing larger viewing angles. Area is also a reasonable metric for accuracy of the geographic collocation, wherein *R_rs_* averaged over larger areas of the ocean may not be representative of those measured at the sampling location, depending on the degree of spatial variability of ocean color within the sampled region. These match-up metrics were described in greater detail in [56].

### 2.4. Statistical Methods

Various metrics and visualization techniques are employed below to gauge the performance of algorithms. Retrieval parameters are compared with the same parameter collected in situ for both the optimization/tuning dataset (i.e., using in situ reflectances) and satellite validation. In addition to common metrics such as the number of samples (N), the standard deviation (STD), and the squared correlation coefficient (r^2^), we evaluate the adjusted r^2^ (r^2’^):

(2) $$r^{2^{\prime }}=r^{2}-\left(1-r^{2}\right)\left(\frac{\beta_{n}}{N-\beta_{n}-1}\right),$$

where *β_n_* is the number of regressors) which adjusts the r^2^ downward to correct for the number of predictive values relative to the number of samples in, for example, multiple linear regression. The root mean square difference (*RMSD*) was also calculated:

(3) $$RMSD=\sqrt{\frac{\sum_{i}^{N}(mod_{i}-ref_{i}{)}^{2}}{N}},$$

where “mod” is the model retrieved parameter and “ref” is the field measurement. The centered-unsigned (or unbiased) *RMSD* (RMSD*′) was defined as follows:

(4) $$RMSD^{*\prime }=\sqrt{\frac{\sum_{i}^{N}(mod_{i}-mean(mod_{i}))-(ref_{i}-mean(ref_{i}){)}^{2}}{N}},$$

and the signed RMSD*′ is simply the RMSD*′ multiplied by the sign of the difference between the *STD* of the model retrieval and the *STD* of the field data (RMSD*′(σ_D_)). The bias and the normalized bias (Bias*) are also employed:

(5) $$Bias^{*}=\frac{\sum_{i}^{N}(mod_{i}-ref_{i})}{N\times STD(ref)}$$

as well as the percent bias (%*Bias*),

(6) $$\%Bias=100\times \frac{mean(mod-ref)}{mean(ref)},$$

and the mean average percent difference

(7) $$\mathrm{MAPD}=100\times mean\left[abs\left(\frac{mod-ref}{ref}\right)\right].$$

While most of these metrics are fairly straightforward, a few warrant further explanation and context. A powerful graphical tool for assessing the skill of model performance—and comparing one model to another—is the Taylor diagram [57], which combines the RMSD*′, *STD*, and correlation into a single figure in which proximity to the field data indicates how well the pattern of the modeled data matches the observations. This is made possible in two dimensions because of the relationship between the RMSD*′, the correlation, and the variances of model and reference. Because the means of the model and reference are removed prior to calculating higher statistics shown in Taylor diagrams, they represent the comparisons between the patterns with any bias removed. For this reason, we have added color here to Taylor diagrams to include %*Bias*. Another graphical assessment used here which accounts for the bias (Bias*) and adds a sign to the RMSD*′ is the target diagram [58], in which the *y*-axis represents normalized bias of the model, the *x*-axis is signed-centered *RMSD*, and distance in any direction from the origin to the model is the total *RMSD*. Here, we also include color in our target diagrams to help visualize the *MAPD*.

## 3. Results and Discussion

### 3.1. Database Characteristics

**Figure 1.**
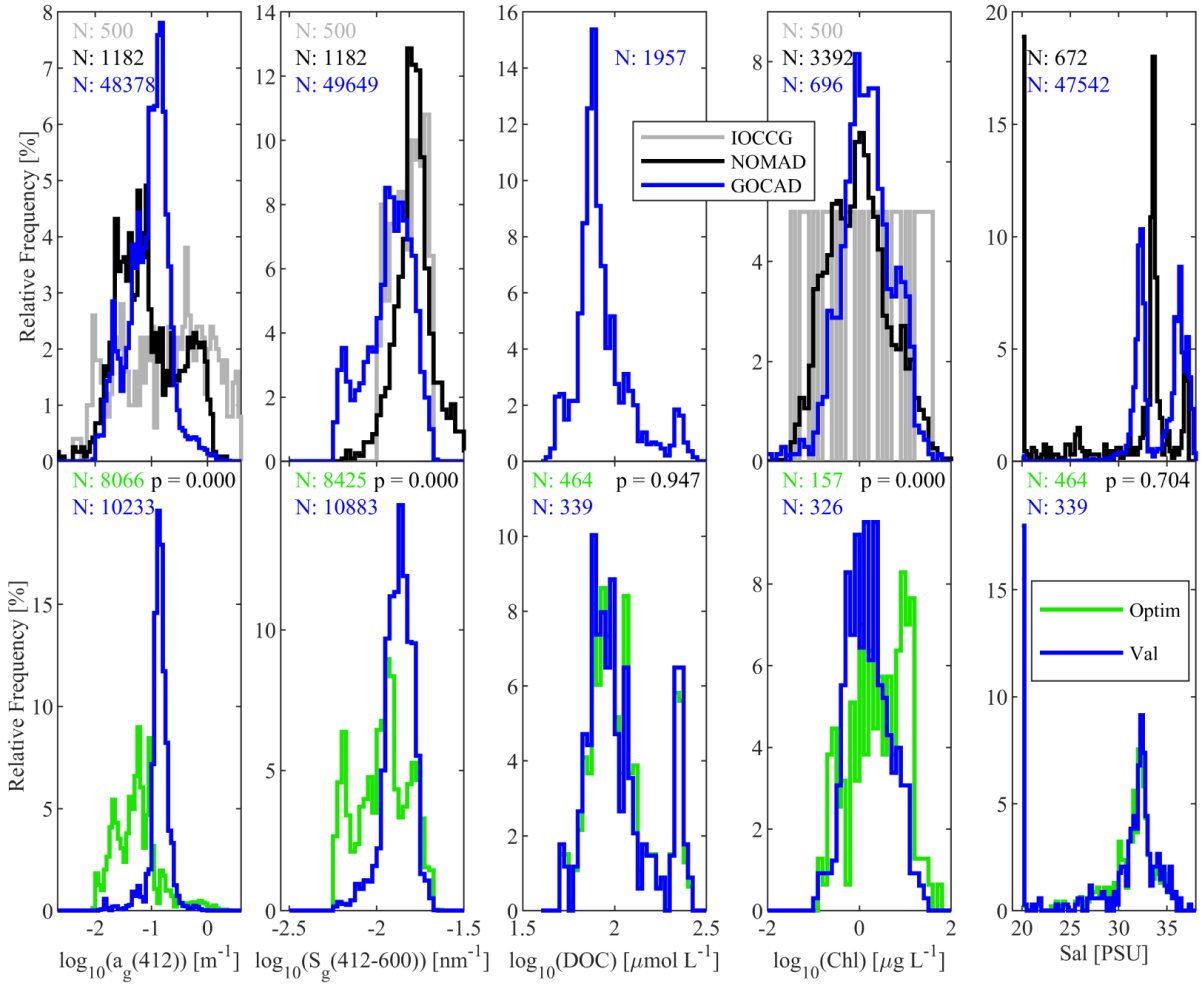
Top row: data distributions and counts (N) of relevant parameters and Chl (for context only) in Global Ocean Carbon Algorithm Database (GOCAD), NASA bio-Optical Marine Algorithm Dataset (NOMAD), and the synthetic ocean color dataset developed by the International Ocean Colour Coordinating Group (IOCCG). Bottom row: comparisons between the subset of GOCAD parameters used in optimization/tuning (Optim) and validation (Val) of algorithms (shown here with SeaWiFS match-ups, but also evaluated for MODIS Terra and Aqua with similar results). Populations of salinity and DOC share a common mean between optimization and validation datasets (ANOVA, *p* > 0.01).

GOCAD has over 40 times more CDOM records than NOMAD, and nearly 100 times as many spectra as IOCCG. It contains *a_g_*(412) data that is more normally distributed than either NOMAD or IOCCG, and a Chl distribution similar to NOMAD (Figure 1). IOCCG model data, while covering the same dynamic range as the two field databases, have unrealistically flat distributions of both CDOM and Chl, raising concerns for introducing bias when the dataset is used for algorithm development and optimization. The dynamic range in *a_g_*(λ) is larger in GOCAD than NOMAD (e.g., *a_g_*(412) from 0.005 to 2.457 m^−1^, and from 0.0013 to 1.923m^−1^, respectively), but the data distribution of GOCAD is narrower than NOMAD and IOCCG (*a_g_*(412) 75th minus 25th percentiles of 0.095 m^−1^, 0.204 m^−1^, 0.627 m^−1^, respectively) around a lower mean absorption level (mean *a_g_*(412) = 0.120 ± 0.133 m^−1^, 0.194 ± 0.266 m^−1^, 0.514 ± 0.745 m^−1^, respectively), reflecting the predominance of low CDOM, offshore data in the database. The large number of field records of CDOM in GOCAD, its range and mean value, all indicate that it is suitable for developing global retrieval algorithms.

The data distributions shown along the bottom row of Figure 1 (with the exception of Chl—see figure caption) show data used to optimize algorithms developed in this study (i.e., from field stations with matching in situ radiometry and IOPs), versus data used for validation—in this case with SeaWiFS wave bands and satellite imagery. For each parameter, distributions of optimization and validation data were compared for similarity to test by analysis of variance (ANOVA) whether the populations share a common mean. Optimization and validation dataset were found to differ (*p* << 0.01) for CDOM absorption and spectral slope, but not for DOC and salinity. The difference between the CDOM and DOC match-up datasets results from availability of the data (i.e., stations may not have both CDOM and DOC measurements in addition to in situ radiometry). Based on the distributions shown in Figure 1, as well as the geographic distributions highlighted in Figure 3, differences between the optimization and validation data populations for CDOM absorption and slope appear to derive from slightly fewer near-shore stations being present in the optimization dataset compared to the validation set, although there is clearly some endmember representation in the optimization set for near-shore conditions. We may conclude from this, however, that algorithms for CDOM absorption and spectral slope developed using these optimization data would perform best in oceanic conditions, while regional algorithms may be more accurate in coastal waters, or waters with very high CDOM absorption and low CDOM spectral slope.

**Figure 2.**
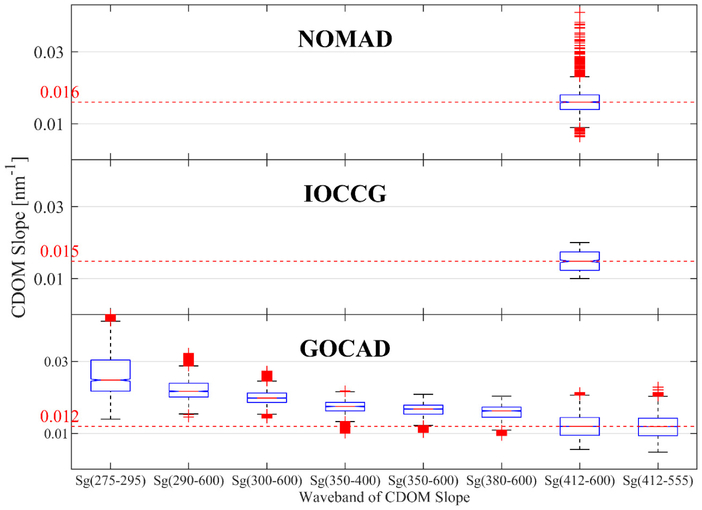
Exponential slope of CDOM in NOMAD, IOCCG, and GOCAD. Median values for S_412–600_ are highlighted in red for comparison. NOMAD and IOCCG lack UV CDOM.

IOCCG and NOMAD contain no UV CDOM data, so direct comparison of spectral slope is only possible in the VIS (Figure 2). The median *S_g_*(412–600) is lower for GOCAD, demonstrating again the predominantly oceanic characteristics (i.e., photodegraded, primarily of marine origin, and presumably refractory) of the CDOM in the database. Slope decreases significantly (*p* << 0.01) as the reference wavelength (i.e., the shortest wavelength in the spectral range) increases from 275–412 nm. Overall, the variability in spectral slope for each range is quite low—generally no more than a factor of 2–3. This narrow dynamic range in slope within each waveband presents a challenge for retrieving fine scale differences in CDOM slope by limiting the sensitivity of algorithms built from inherently uncertain ocean color. However, errors in the retrievals should be small relative to the absolute magnitude of the slope even if the algorithm sensitivity (e.g., correlation between retrievals and field measurements) is low.

**Figure 3.**
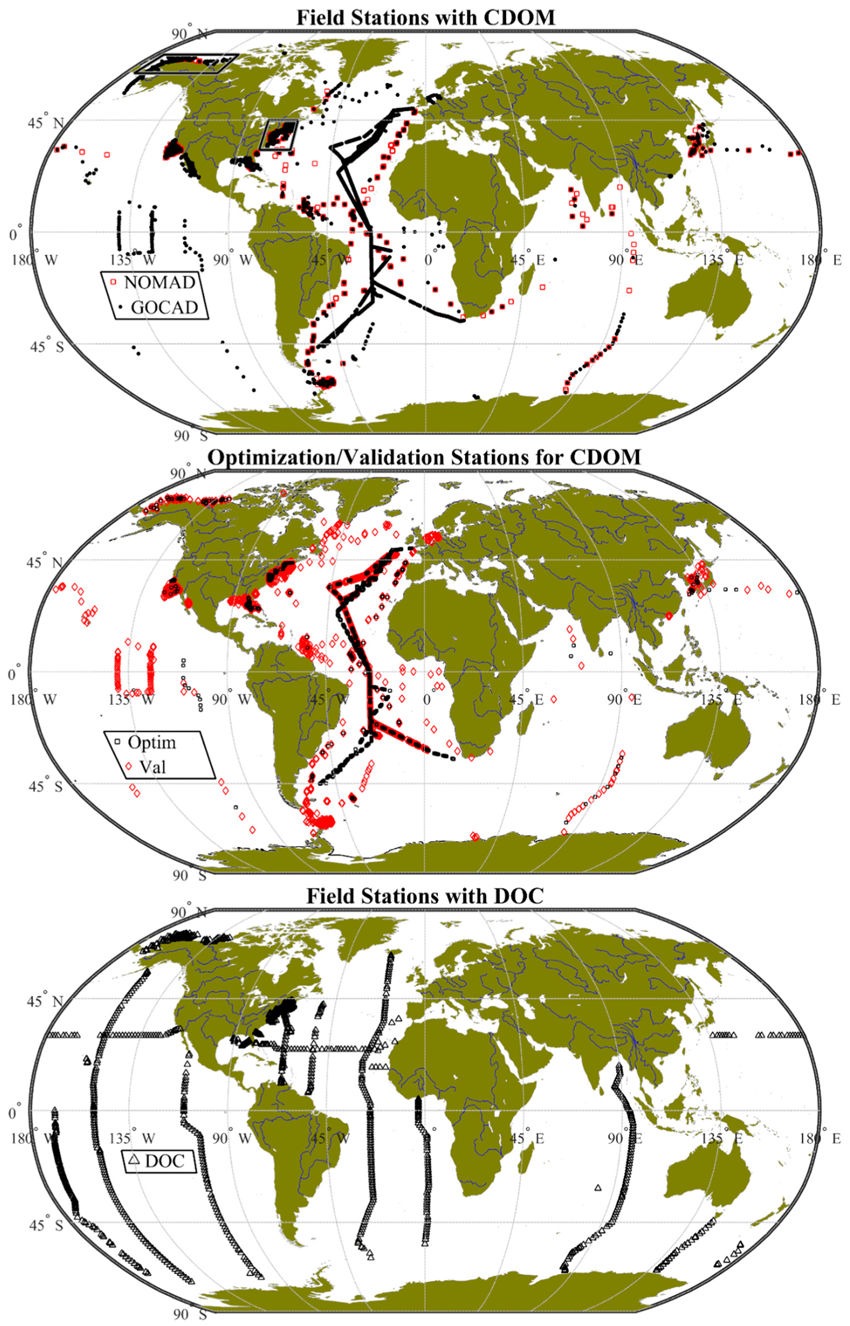
Global distribution of GOCAD and NOMAD field stations for CDOM (upper) and DOC (lower). The central panel shows the distributions of data within GOCAD separated into optimization (Optim) and validation (Val) dataset. Stations used in algorithm tuning are shown as red circles, the remainder of stations were available for satellite validation. The boxed subregions in the upper panel are shown in greater detail in Figure 4.

Geographic locations of GOCAD field stations overlap with NOMAD stations (Figure 3). We can see that many of the NOMAD stations were ultimately excluded from GOCAD during the quality assurance analysis described in Section 2.2. Highlighted in the central panel of Figure 3 are those stations with high-quality in situ radiometry, which were set aside for tuning, training, and optimization of ocean color algorithms. The geographic distributions of both the training data and the validation data show a representational combination of stations from both offshore and nearshore waters, which theoretically improves the odds of being able to retrieve a broad dynamic range of bio-optical properties, although as pointed out above the optimization data appears to be slightly dominated by oceanic stations. It is clear from Figure 3 that while there is significant overlap in the CDOM and DOC datasets in certain regions such as northern Alaska and the mid-Atlantic Bight in the Northeastern U.S., globally they follow a somewhat different pattern, and many DOC field stations are not obviously represented in the CDOM dataset.

**Figure 4.**
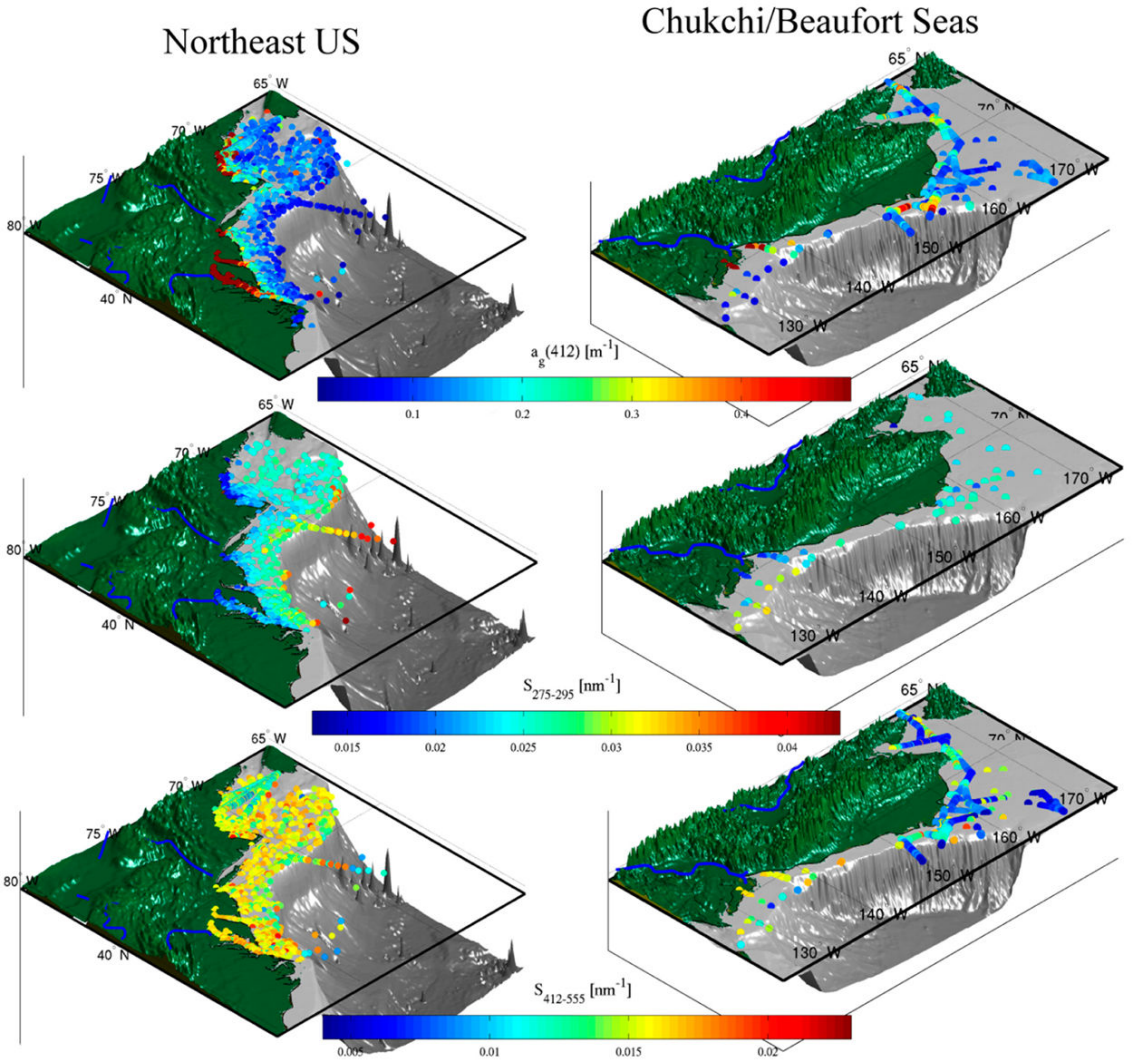
Examples of CDOM absorption at 412 nm (top row), and CDOM spectral slope in the UVB (middle row) and VIS (bottom row) from GOCAD show patterns which reflect the sources and age of CDOM in environments stretching from estuarine, such as the Chesapeake Bay in the eastern U.S., to stations sampled well offshore.

The dense concentrations of field stations sampled in relatively smaller regions such as the Northeastern U.S. are difficult to resolve at the small scale in Figure 3. Figure 4 shows three-dimensional maps of select sub-regions with *a_g_*(412), *S_g_*(275–295), and *S_g_*(412–555), including the Northeast US and coastal Alaska between the Chukchi Sea and the Beaufort Sea. These are set in broad continental shelves with numerous nearby river outflows. Not surprisingly, CDOM is high throughout the regions shown in Figure 4 with low spectral slope in the UV. CDOM and *S_g_*(275–295) increase and decrease, respectively, in close relation to distance from shore, as expected given the considerations discussed in Section 1.2 and elsewhere. Variability is higher for spectral slope in the VIS (*S_g_*(412–555)), but it generally follows the opposite pattern from that in the UV, i.e., decreasing with distance from new sources of CDOM. This is indicative of aging processes as the newly mobilized, near-shore CDOM mixes seaward and photo- and microbial degradation reduce absorption in the UVA relative to the UVB (thus increasing *S_g_*(275–295) and relative to the VIS (thus decreasing *S_g_*(412–555)). It may indicate marine sources of CDOM with chromophores that absorb in the UVA and blue rather than terrestrial sources that also absorb in the UVB. These patterns are perhaps clearest at the outflows of the Colville River (~135° W and 70° N) and the Chesapeake Bay (~77° W and 37° N). An interesting exception for *S_g_*(412–555) can be found in the Gulf Stream transect (~70° W and 37°M0° N; GOMECC-2 experiment, Table 2), where slope increases upon entering the productive waters at the edge of the Gulf Stream despite a lack of CDOM increase, and then rapidly declines upon entering the oligotrophic waters south of the Gulf Stream.

### 3.2. Algorithm Tuning and Validation

#### 3.2.1. Algorithm Structures and Optimization

**Figure 5.**
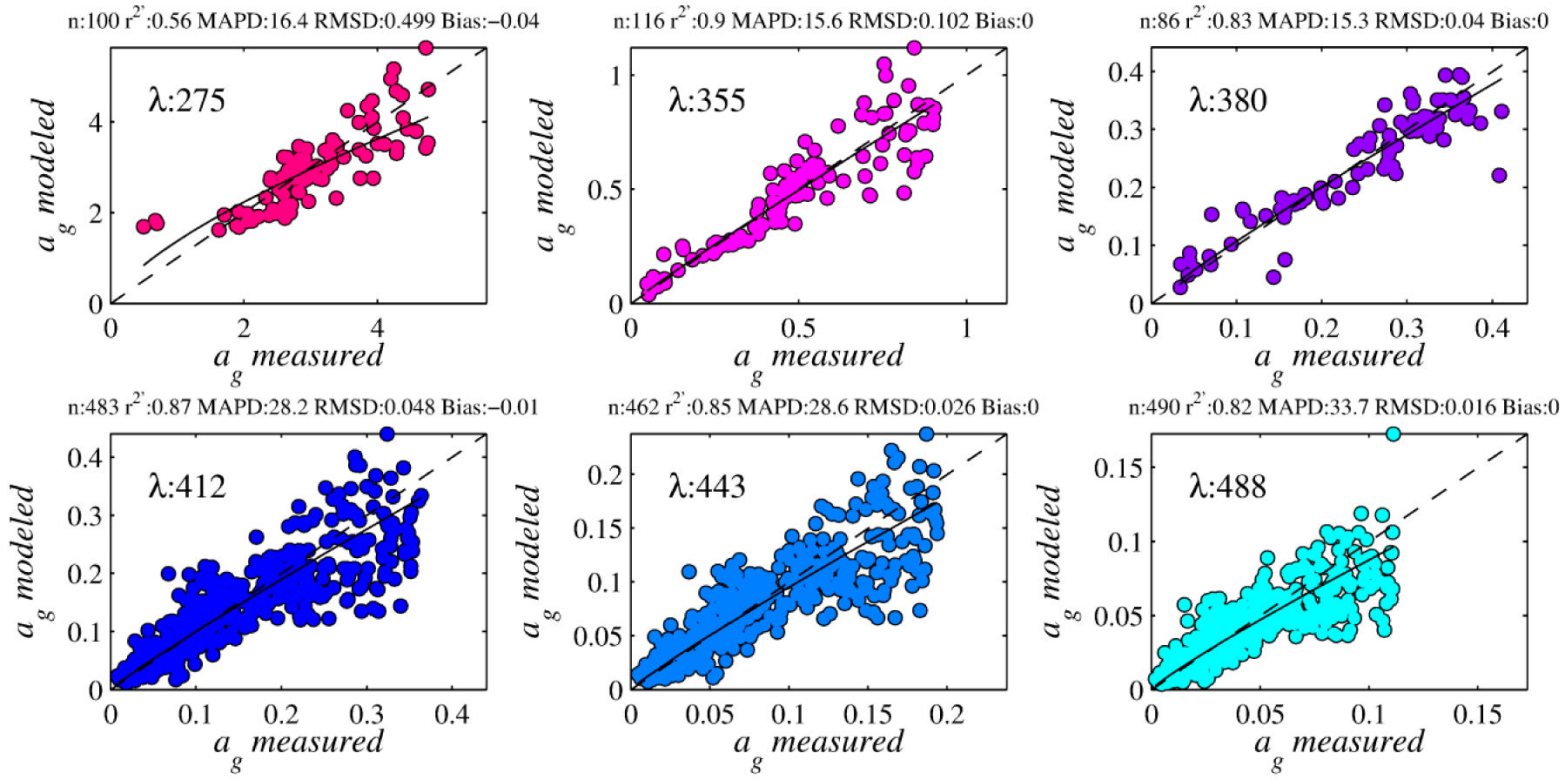
MLR retrievals of CDOM plotted against field data for the tuning dataset (i.e., in situ *R_rs_*(*λ*)). The solid line shows the fit through the data, and the 1:1 line is dashed.

Both empirical and semi-analytical approaches to ocean color retrievals of CDOM, *S_g_*, and DOC were explored using the GOCAD dataset. Of the former, a band ratio, single exponential decay model similar to that presented by Mannino (2008) was tested, but found to be better suited for the continental shelf waters for which it was derived rather than for the deep ocean, and will not be presented here. A multiple linear regression (MLR) approach was tested matching the natural logarithm of *R_rs_* in four ocean color bands with the logarithm of *a_g_*(*λ*) and *S_g_* at each waveband described in Section 2.2.1, and DOC. The least square difference minimization regression, performed using Matlab’s *regstats* function (www.mathworks.com), follows the form:

(8) $$\text{ln}(\Upsilon )=\beta_{0}+\beta_{1}\times \text{ln}(R_{rs}(\lambda_{1}))+\beta_{2}\times \text{ln}(R_{rs}(\lambda_{2}))+\beta_{3}\times \text{ln}(R_{rs}(\lambda_{3}))\;\;+\beta_{4}\times \text{ln}(R_{rs}(\lambda_{4}))$$

where *β*_0_–*β*_4_ are the regression coefficients, *γ* is the retrieval parameter, and *λ_1_*–*λ_4_* are the sensor-specific wavelengths (i.e., 443, 488, 531, and 547 nm for MODIS, 443, 490, 510, and 555 nm for SeaWiFS). Using monthly, binned L3 Aqua imagery for 2010, MLR retrievals were used to establish the 99th percentiles for each retrieval waveband of *a_g_*. Retrievals above these values were considered outside the scope of this global algorithm, and eliminated. Regression coefficients, statistics, and thresholds are presented in Figure 5, and Tables 3–5. Model retrievals plotted against field data are well organized about the 1:1 line with low scatter, particularly in the UVA, which is reflected in high correlation coefficients and low error and bias. *MAPD* is below 30% for all bands below 488 nm; from this band to higher wavelengths, the CDOM signal becomes very weak in most of the global ocean.

For the reasons outlined in Section 1.4 (i.e., a large and variable portion of DOC is unpigmented), DOC derived directly from ocean color alone using MLR was not robust (Table 5; MLR1). However, satellite retrievals of sea surface salinity are now available thanks to the Aquarius mission (http://aquarius.nasa.gov/), and for CDOM, salinity was a reasonable choice as an additional proxy for water type considering it will generally reflect proximity to sources of fresh water and CDOM as well as distinguishing water masses (e.g., Gulf Stream). Using GOCAD, a multiple linear regression approach was developed for retrieving DOC from *a_g_*(355) (in place of *R_rs_*(*λ*_1_) in Equation (8)) and salinity (in place of *R_rs_*(*λ*_2_)), and proved very robust (e.g., r^2^ = 0.91, %*Bias* = 0). Using CDOM and salinity as predictors significantly improved retrievals of DOC (Table 5; MLR2), with r^2′^ increasing from 0.76 to 0.91, and *MAPD* dropping by about three percentage points. The strength of the correlation between field and retrieved DOC to CDOM and salinity is stronger than expected, considering the many ways in which changes in DOC, CDOM, and salinity may diverge across seasons or from region to region. It is worth pointing out that other factors may be contributing to the stronger statistical performance of MLR2 over MLR1, such as the higher number of coincident predictors and retrievals, as well as the absence of uncertainties associated with reflectance data in the tuning dataset (i.e., DOC is derived directly from CDOM absorption and salinity). Caution is therefore advised when applying this DOC algorithm in regions in which DOC is known to change without commensurate changes in CDOM and/or salinity. For example, the accumulation of DOC in surface subtropical waters including the BATS field station [59,60] appears to be decoupled from CDOM (Norman Nelson and Craig Carlson, personal communication).

**Figure 6.**
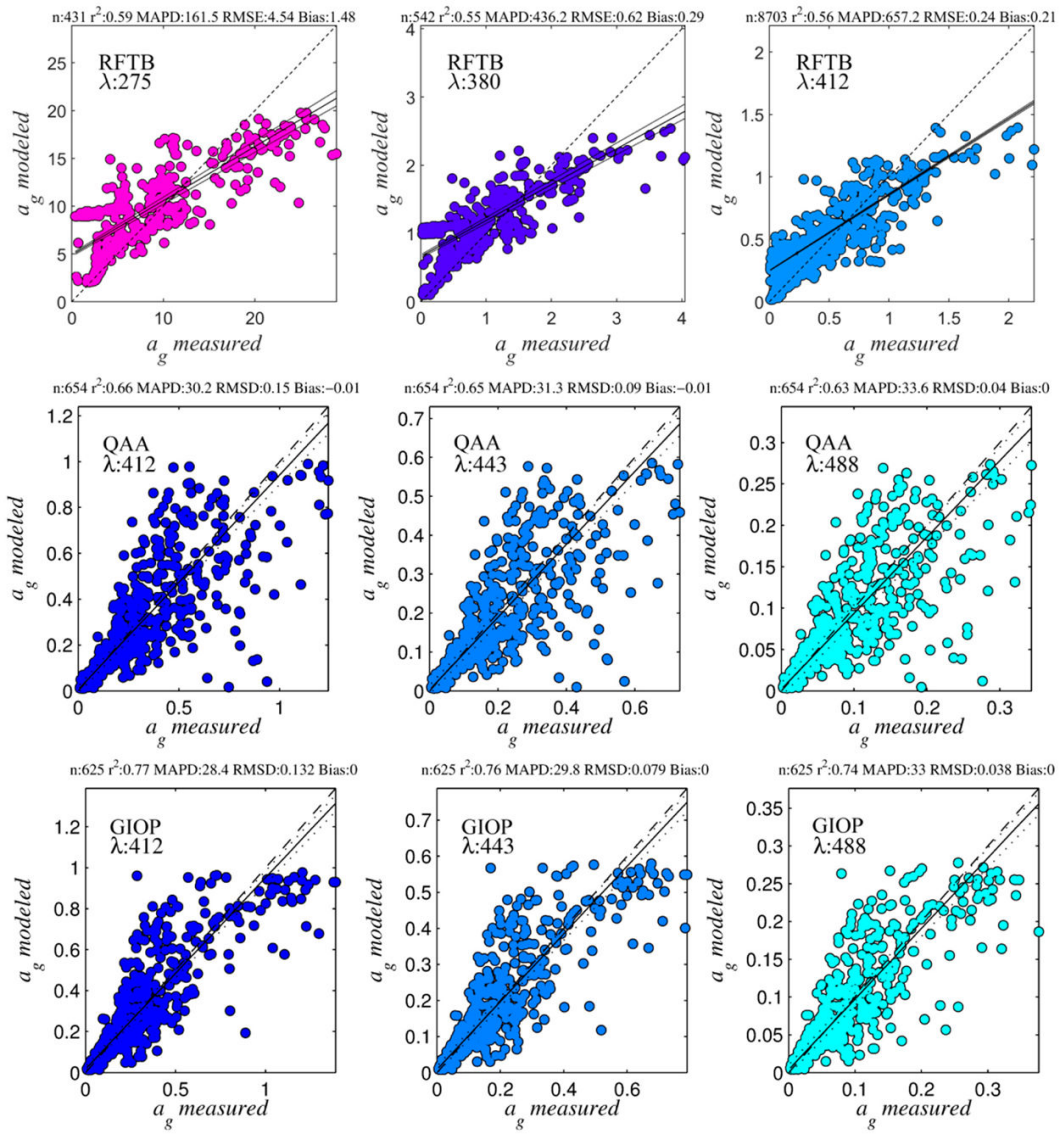
Random forest tree-bagger (RFTB), quasi-analytical algorithm (QAA), and generalized inherent optical property (GIOP) retrievals for tuning datasets.

Another empirical approach tested here was the machine learning approach known as Random Forests [61,62], which is a method for multivariate, non-linear, non-parametric regression designed to help minimize over-fitting of the training dataset. The method improves on standard decision tree regression performance by using an ensemble of independent decision trees; bootstrapping for the regression is achieved by repeatedly, randomly resampling the original dataset to provide an ensemble of smaller independent datasets, which are each used to grow a decision tree (hence the term random forest tree-bagger, or RFTB). Here, 200 independent decision trees were used, and each tree is trained on approximately 66% of the training dataset. The inputs (i.e., reflectances) and retrievals of the regression (i.e., CDOM, CDOM slope, and DOC) were the same as in the MLR. Model performance and statistics for select bands in the UV and VIS with the training dataset are presented in Figure 6. Comparisons of model retrievals to field data are fairly well correlated, but error and bias are quite high, with *MAPD* reaching several hundred percent.

Semi-analytical approaches included the Quasi-Analytical Algorithm of (QAA) [5,63,64], and the Generalized Inherent Optical Property (GIOP) algorithm [65]. These have the advantage that they are based on theoretical models for how the light field is affected by the inherent properties of the water, but can only retrieve IOPs at those wavebands for which *R_rs_*(*λ*) is measured (i.e., they do not extend into the UV for the current and historical suite of satellite sensors). In the future, however, data from GOCAD and elsewhere could be used in the development of linear matrix inversion-type semi-analytical algorithms including basis vector models extending into the UV for CDOM, thereby potentially enabling their retrieval directly using SAAs. In fact, using GOCAD to build more globally representational basis models extending CDOM into the UV may not only provide better retrievals of CDOM, but also of the other concurrently retrieved optical properties from linear matrix inversion. Both the GIOP and QAA invert the *R_rs_*(*λ*) to retrieve the water column IOPs following the theory that sea surface reflectance at a given wavelength is proportional to the backscattering coefficient, and inversely proportional to the absorption coefficient [16,66]. Each uses various assumptions, empirical parameterizations, and mathematical inversion techniques to solve for the IOPs and partition them into their constituents. These include the total backscattering coefficient, *b_bt_*(*λ*), backscattering by particles, *b_bp_*(*λ*), absorption by total particles, by phytoplankton, and by the combination of non-algal particles and CDOM, *a_dg_*(*λ*) = *a_d_*(*λ*) + *a_g_*(*λ*), where *a_d_*(*λ*) is non-algal (or detrital) absorption). These latter properties are similar in spectral shape, and therefore difficult to partition, which presents a challenge if we wish to compare the retrievals of SAAs to the other algorithms presented here. Therefore, while we do not re-develop or re-optimize the SAAs here—using them as published—we do utilize GOCAD to facilitate the separation of dissolved and detrital absorption components. Specifically, we solve for *a_g_*(*λ*) by assuming that *a_d_*(*λ*) is a function of the combined backscattering by water, non-algal particles, and the dissolved absorption by CDOM, which we assume does not backscatter, although there is some evidence supporting backscattering by colloids [46]. These SAAs retrieve only the combined *b_bp_*(*λ*) from phytoplankton and non-algal particles, but the latter tend to have a higher refractive index, and therefore contribute far more strongly to the backscattering signature, e.g., [67] and references therein. An empirical relationship was developed between *a_d_*(410), *b_bt_*(550) and *a_dg_*(410), and then *a_g_*(410) was found by subtracting *a_d_*(410) from SAA retrievals of *a_dg_*(410):

(9) $$a_{d}(410)=0.06822\times a_{d_{g}}(410)+1.623\times b_{bt}(550)+0.0002123$$

Due to a paucity of *a_d_*(*λ*) and *b_bt_*(*λ*) observations in GOCAD, this relationship was tuned for multiple linear regression using the IOCCG synthetic dataset (r^2′^ = 0.76, *RMSD* = 0.07, bias = −0.004 m^−1^, *MAPD* = 75%, N = 464). *a_g_*(410) was expanded using Equation (1) to other wavebands with the empirical retrieval for *S_g_*(412–555) (derived as per Equation (8) and Table 4). Regression statics for the optimization data are shown for the QAA and GIOP in Figure 6, with slightly better results in the GIOP. Although the current version of GOCAD is less well populated with some optical properties than others (i.e., data collection targeted carbon-related properties and only included others if they happened to be in the same SeaBASS file), the digital structures for each property mentioned in this section are included in the database, and future algorithm investigation (particularly using SAAs) would greatly benefit from incorporation of these data into GOCAD or a similar, climate-scale, global database.

#### 3.2.2. Algorithm Validation

This work represents the most rigorously validated set of global CDOM and DOC algorithms to date. Optimization/training of algorithms as described in the previous section was conducted on GOCAD field stations with coincident in situ radiometry. These stations were then set aside from validation, which was performed only on those remaining stations in GOCAD that had coincident satellite imagery (i.e., MODIS Aqua, Terra, and SeaWiFS). In addition to the algorithms already mentioned, two other empirical algorithms based on band ratio approaches were included in validation analysis. The approach of Shanmugam (2011) [31] (hereafter Shan11) used a power-law relationship between the ratio of *R_rs_*(443)/*R_rs_*(555) and *a_g_*(350) and *a_g_*(412), and performed well using the NOMAD dataset. The ratio of these was then used in another power-law function to solve for *S_g_*(350–412). Tiwari and Shanmugam (2011) [32] (hereafter TS11) used linear functions to relate the ratio of *R_rs_*(670)/*R_rs_*(490) to *a_g_*(412) and *a_g_*(443), and solved for *S_g_*(412–670) analytically by inverting Equation (1). As these two algorithms were tuned using SeaWiFS bands, a slight adjustment was made to MODIS input reflectances to obtain the SeaWiFS reflectances required (only MODIS validation is shown here graphically).

**Figure 7.**
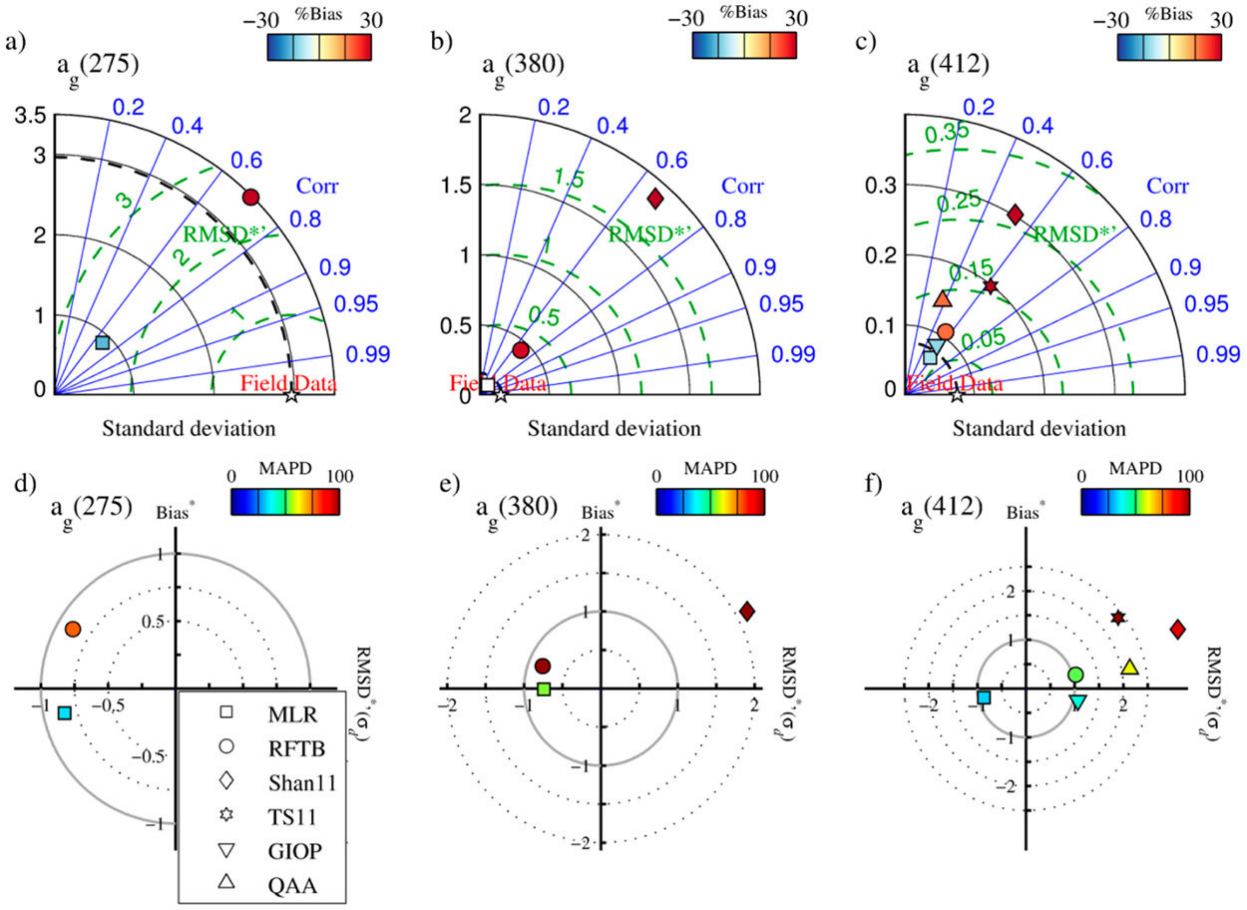
Taylor diagrams (top row) and target plots (bottom row) depicting comparative algorithm performance for retrieving CDOM absorption at 275 nm, 380 nm, and 412 nm from MODIS Aqua.

**Figure 8.**
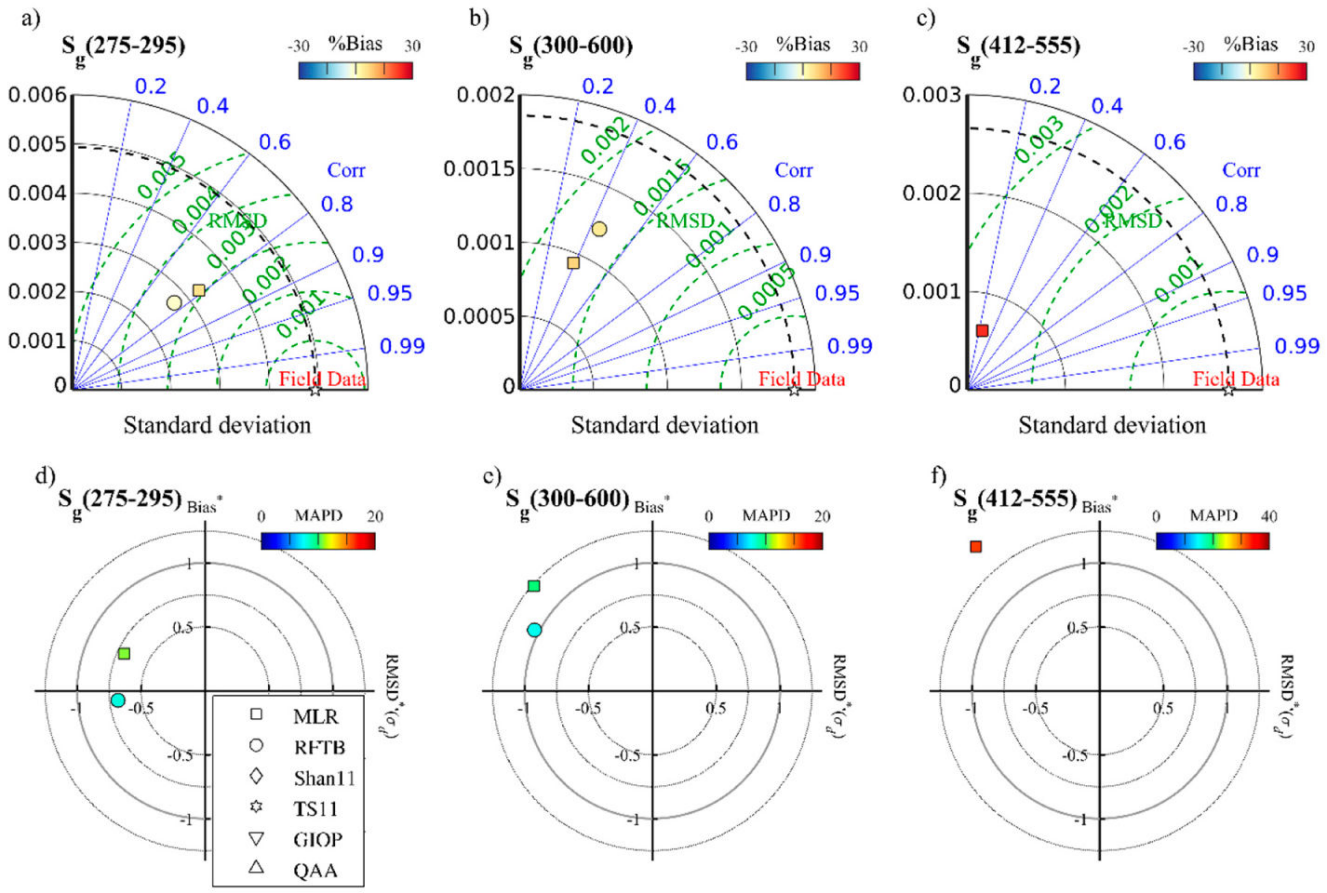
Taylor diagrams (top row) and target plots (bottom row) depicting comparative algorithm performance for retrieving CDOM slope at 275–295 nm, 300–600 nm, and 412–600 nm from MODIS Aqua. Results from Shanll and TS11 were suppressed to preserve scale.

**Figure 9.**
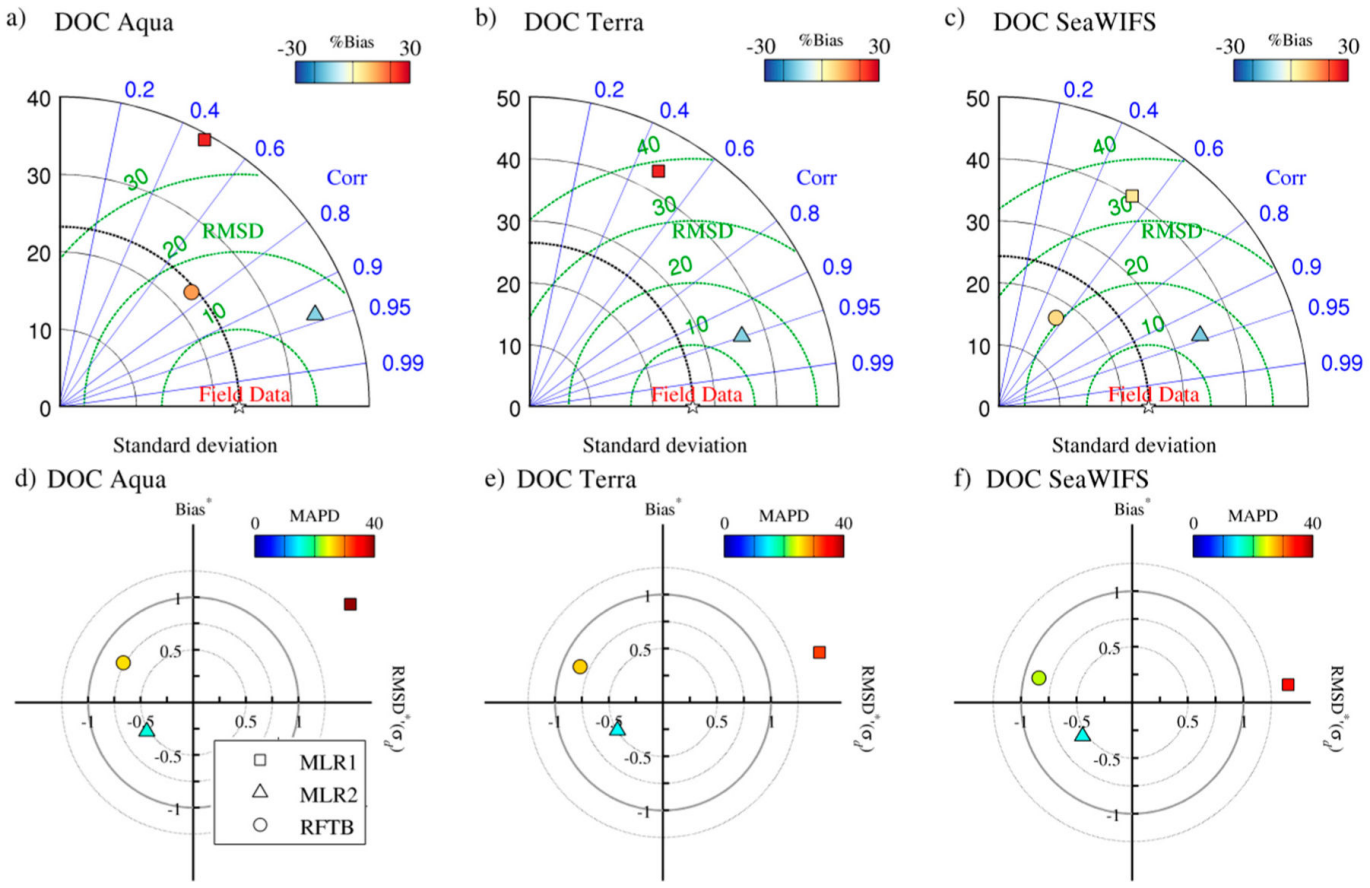
Taylor diagrams (top row) and target plots (bottom row) depicting comparative algorithm performance for retrieving DOC from MODIS Aqua and Terra, and SeaWiFS.

The performance of all algorithms in independent validation is weaker than for optimization (Tables 6–8, Figures 7–9). This should not be surprising considering satellite imagery is subject to higher uncertainty associated with atmospheric correction, where the atmosphere comprises ~90% or more of the signal received by the satellite sensor. Furthermore, satellite match-ups exacerbate the issue of temporal and geographic coincidence with in situ measurements. Any regions of moderate to high variability in surface properties will likely not be well captured by the average of a nominally 5 km × 5 km pixel array. Nevertheless, results are encouraging, particularly for the MLR approach and particularly in the UV, where the CDOM signal is strongest (in terms of in situ data) and the SAAs are not currently useful.

Figure 7 shows Taylor and target diagrams comparing the CDOM absorption retrieval metrics for various algorithms as described in Section 2.4. In the UVB (275 nm), and UVA (380 nm), only the empirical approaches were feasible, while SAAs (i.e., QAA and GIOP) are also shown at 412 nm. MLR and RFTB perform comparably with respect to correlation between the models and measurements at 275 nm, although MLR does have significantly lower *MAPD* and bias (Table 6; MLR highlighted in bold), and outperforms the RFTB at 380 nm in all but correlation for all sensors. MLR also outperforms all other CDOM absorption algorithms at 412 nm, although GIOP does not appear significantly worse as seen by its proximity to field data in the Taylor plots and the origin in the target diagrams. MLR shows a relatively strong negative bias in most sensors and channels, which is the result of underestimation in high CDOM waters (data not shown).

CDOM spectral slope was only retrievable with empirical approaches. MLR and RFTB performed comparably to each other, although RFTB was not tested at *S_g_*(412–555). In the application of retrieval algorithms for *S_g_* below (Section 3.3), the MLR is used mainly for its simplicity, but we would expect RTFB retrievals to yield nearly equally accurate results. Shan11 and TS11 performed poorly (Table 7; MLR highlighted in bold). Correlations between modeled and measured CDOM slope were weak in the UVA and VIS, but as the dynamic range of the field data is quite low (Figure 2), error and bias were still low in the retrievals (Table 7). In all sensors and bands for the MLR and RFTB, *S_g_* tends to be slightly overestimated in waters with low *S_g_*, and slightly underestimated in waters with high *S_g_*, indicating the weak sensitivity of these empirical approaches also reflected in the low correlation coefficients.

As anticipated, MLR retrievals of DOC using ocean color alone were only weakly correlated with field data (i.e., r^2^ < 0.3, Table 8). RFTB performed considerably better, but was unable to match the performance of MLR2 (i.e., regression with retrievals of *a_g_*(355) and known salinity; highlighted in bold in Table 8). Due to the newness of the Aquarius mission, there were too few retrievals available for incorporation in these validation results, and further validation of this approach is encouraged based on these results.

**Figure 10.**
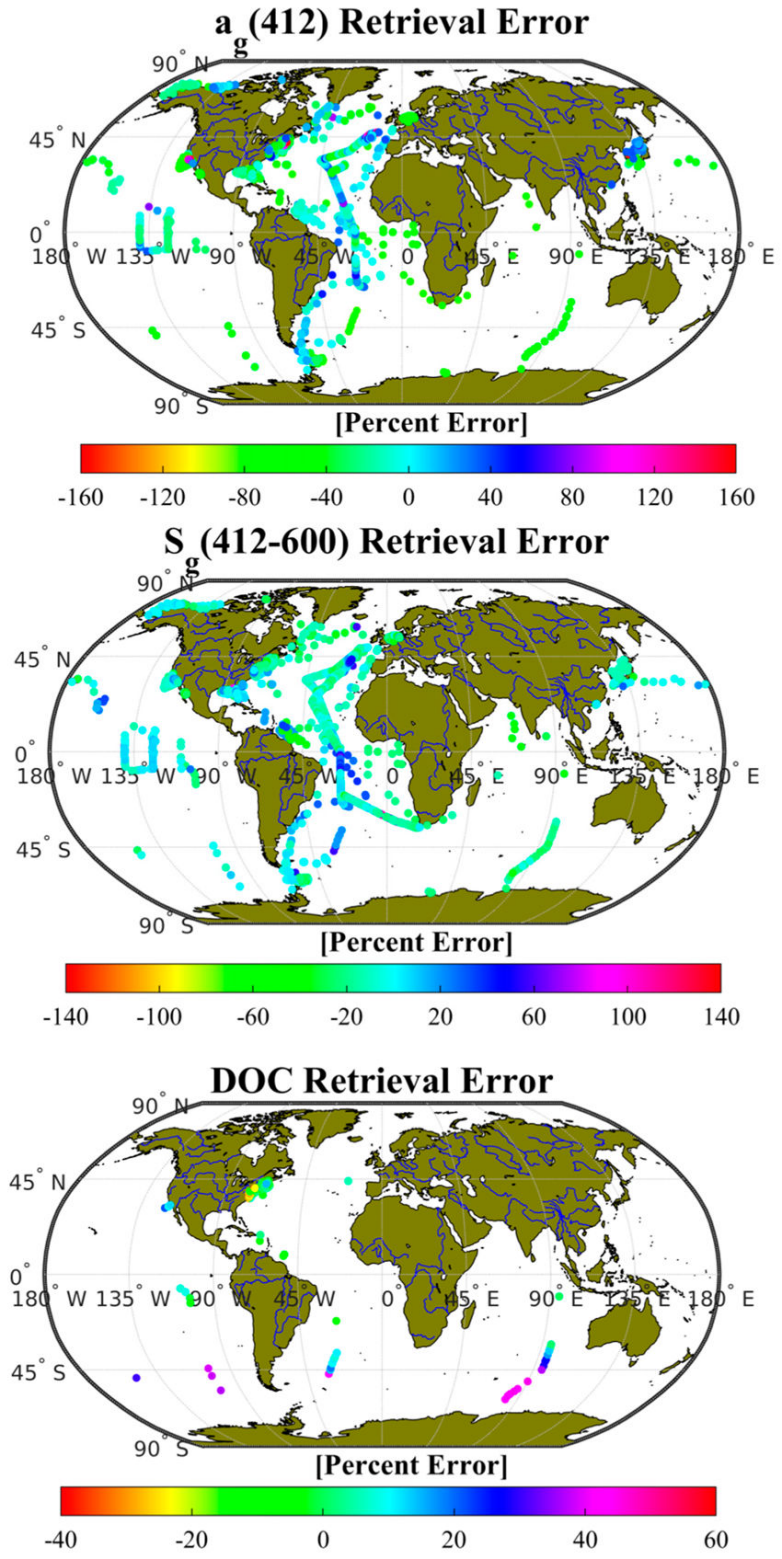
Geographic distribution of error in MLR algorithm retrievals of CDOM absorption and slope in the VIS (top and center), and MLR2 retrievals of DOC (bottom) using validation stations and satellite imagery.

We speculated above (Section 3.1) that small differences between the optimization and validation records may bias algorithm performance to favor oceanic waters. To test this hypothesis, a sensitivity analysis was evaluated for CDOM absorption retrieval by MLR to test correlations between algorithm error (percent error between retrievals and field data) and salinity, water column depth, and *a_g_*(412) measured in the field. We found no sensitivity to these factors (r^2^ < 0.04 in each case, n = 29,757 for Aqua, Terra, and SeaWiFS combined), indicating that the algorithm is not optimized in a way that would limit its performance in, for example, high salinity, offshore waters, or fresher waters with high inputs of fresh CDOM. A geographic distribution of algorithm retrieval error (percent error) for *a_g_*(412) and *S_g_*(412–600) is shown in Figure 10.

A similar sensitivity analysis was evaluated for MLR2 (DOC retrieval) performance at validation stations to help identify limitations of the algorithm. We tested the correlation between the DOC retrieval error (percent difference between retrieved and measured DOC) and salinity, water column depth, and DOC concentration, but found no strong trends in the distribution of error (r^2^ = 0.17, 0.19, 0.43, respectively, see included figures below), although it could be argued that absolute retrieval error increases somewhat (overestimates) at the extremely high salinity stations, and at extremely low DOC stations. In general, it appears that shallow stations underestimate DOC, and deeper stations tend to overestimate. The geographic distribution of error in algorithm retrievals (Figure 10) revealed no patterns with respect to distance from shore or nearby fluvial sources, but MODIS Aqua retrievals did overestimate DOC in southern oceans (south of 40° S, 41% ± 16%, n = 18) compared to minor underestimates from other sensors and at latitudes north of 40° S (—8% ± 18%, n = 1054). Care should therefore be taken when evaluating algorithm retrievals in these areas. Only 1,090 stations (all sensors combined) were available for validation of the MLR2 and this analysis of sensitivity, and their distribution is not uniform across the world’s oceans, but, as shown in Figure 1, a broad spectrum of water types with a large dynamic range of DOC were represented in both the optimization and validation datasets. Unfortunately, no validation stations for MLR2 were identified for mid-ocean gyres, and therefore the performance of the MLR2 in those waters remains poorly defined, and caution is advised in the interpretation of DOC retrievals in those areas.

Differences between retrieval statistics across satellite platforms using the MLR approaches were generally small, with Aqua and Terra outperforming SeaWiFS for CDOM absorption (Table 6). All three sensors performed comparably for *S_g_* and DOC (Tables 7 and 8).

### 3.3. CDOM, S_g_, and DOC Climatology

**Figure 11.**
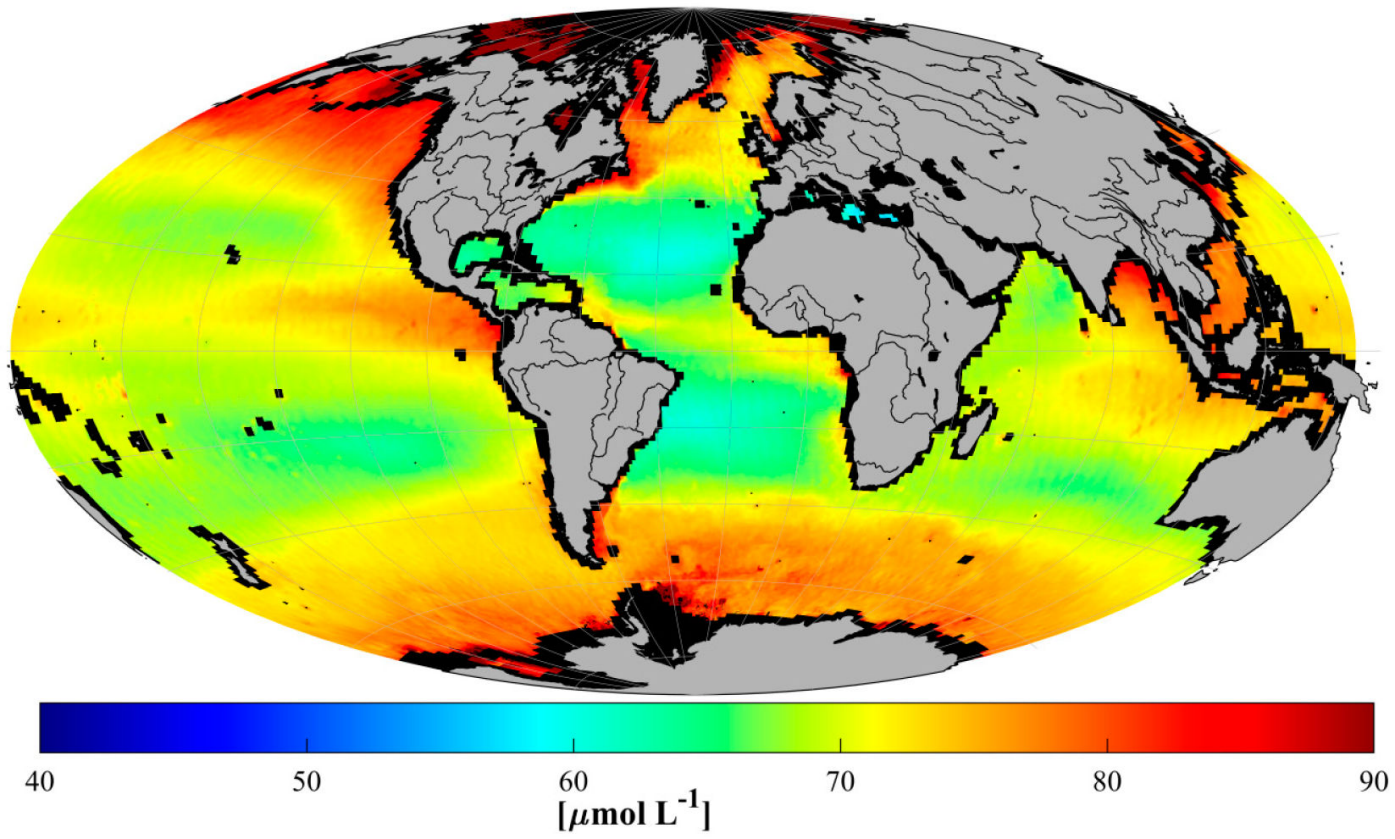
Retrieved three-year mean, 9 km nominal resolution DOC from Aquarius and MODIS Aqua using the MLR2 inversion. Validation statistics are reasonably good for the MLR2 (Figure 9, Table 8), but a larger number and wider geographic distribution of validation stations than are currently available is required to thoroughly evaluate the geographic and water-type limitations for MLR2, particularly in the mid-ocean gyres (see text Section 3.2.2). Overestimates of DOC (~41%) retrieved with the MLR2 were found in the southern oceans (S of 40° S)), but only for MODIS Aqua (i.e., not Terra, and no SeaWiFS stations were identified). Elsewhere (i.e., north of 40° S), retrievals tend to slightly underestimate DOC (<10%). Caution is therefore advised in interpreting MLR2 retrievals in mid-ocean gyres, and in the southern oceans using Aqua.

Because the Aquarius mission (providing sea surface salinity) was limited to <4 year data record (~August 2011–June 2015), climatologies for retrieved DOC similar to those presented below for *a_g_* and *S_g_* are not possible. Instead, three years (2011–2013) of coincident MODIS Aqua and Aquarius data were used to generate a three-year mean 9 km global DOC product (Figure 11). An overlay of in situ surface DOC from GOCAD was examined, but not included here because with no temporal coincidence in this comparison, strong biases likely to occur in the field data (e.g., field sampling of high latitudes is proscribed during winter for obvious practical reasons) will not be reflected in the mean DOC satellite product. Nevertheless, the relatively large (±~50%–~100%) disparities apparent in several regions—including high latitudes and the Atlantic subtropical gyre—indicate fundamental weaknesses in the global DOC algorithm. For instance, as mentioned in 3.2.1, the subtropical Atlantic gyre is characterized by an accumulation of DOM not reflected in the CDOM nor apparently traceable with changes in salinity, and is therefore overlooked by the DOC algorithm presented here (MLR2). Based on the tuning statistics, there appears to be merit in the approach, but more study will be required to establish when and where the algorithm works best, and what (if anything) can be done for remotely sensing DOC in regions where no robust optical proxies exist.

As outlined in the introductory sections, a common assumption made in ocean color remote sensing on a global scale is that CDOM and other water-borne pigmented material covary with Chl. A valid concern with empirical approaches to retrieve CDOM from ocean color—particularly for those that use some of the same spectral bands as Chl algorithms like OC3M—is that they are essentially tuning themselves to Chl, and not CDOM. While performance metrics for MLR are quite robust (e.g., Figure 5, Table 3), there remains the possibility that this is true in part because these spectral bands are sensitive to Chl, and CDOM is simply covarying with Chl as per Case 1 assumptions. In fact, this was not found to be the case generally from global CDOM and Chl field data in GOCAD (r^2^ = 0.00, N = 19,446, λ = 412), nor in the investigation by Siegel et al. (2002), although there exist areas of open ocean outside of the strong influence of terrestrial run-off and upwelling zones where fluctuations in CDOM are clearly driven by local productivity.

**Figure 12.**
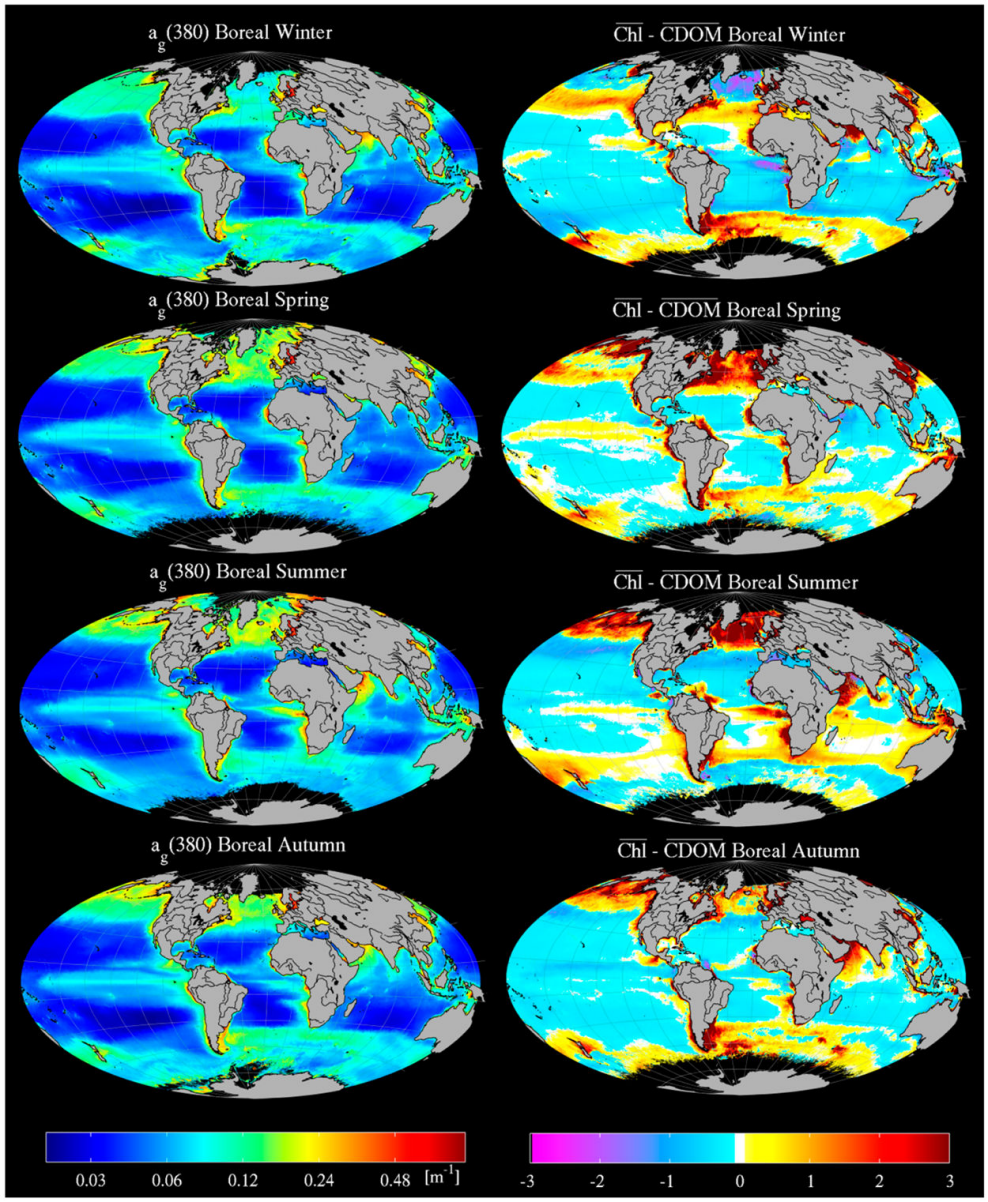
MLR retrievals of *a_g_*(380) by season over the entire MODIS Aqua era (left column), and residuals between Chl and CDOM (right column). Imagery was binned from 4 km resolution monthly composites between August 2002 and January 2014.

To quantify the distinction between retrievals of CDOM using MLR and Chl using OC3M, we calculate retrievals of each over the course of the entire MODIS Aqua mission, and then calculate a residual of the normalized properties, as defined by:

(10) $$\bar{Chl}-\bar{CDOM}=\frac{Chl_{i}}{median(Chl)}-\frac{a_{g_{i}}(\lambda )}{median(a_{g}(\lambda ))}$$

where the median is taken over an entire composite image to scale each property by its magnitude. For example, MODIS imagery was separated into seasonal composites for the entire Aqua era, then processed to CDOM and Chl, and the residual calculated for each (Figure 12). Bear in mind in this analysis that OC3M-like algorithms for retrieving Chl have been shown to be strongly influenced by not only phytoplankton biomass, but also physiology (particularly in tropical and subtropical regions) as well as the presence of significant absorption by CDOM and non-algal particulates (*a_dg_*) [68].

The results show that over much of the world’s oceans—particularly at high latitudes, upwelling zones, and regions influenced by large river plumes—normalized CDOM and Chl diverge by as much as a factor of three. Interestingly, the regions shown by Siegel et al. (2013) to be most negatively impacted by *a_dg_* in terms of empirical Chl retrievals are the same regions which are shown here to diverge most strongly in terms of the normalized CDOM and Chl residual, indicating that a similar pattern would be expected even when using Chl retrieval algorithms less susceptible to error induced by *a_dg_*. The pattern that emerges is that in open ocean regions characterized by strong seasonal blooms such as the North Atlantic and North Pacific, high primary productivity in the presence of lower CDOM (i.e., high residual) is followed after approximately a season by higher CDOM and a collapse in Chl (i.e., low residual). This can be seen in the boreal Spring–Summer transition in the N. Atlantic and Pacific, in the bloom and collapse associated with the reversal of the monsoons in the Arabian Sea between Summer and the following Winter/Spring, and in the Congo and Amazon river plumes over the same period where the residual often shifts by approximately a factor of six between seasons. This observed seasonal lag between peak Chl and peak CDOM helps explain why the two properties rarely covary, as described above for GOCAD, and in [13]. The lag may be explained by the time required for microbial degradation of the bloom’s less labile particulate detrital material after the bloom has collapsed.

Application of these algorithms also shows the Spring–Summer transition in the CDOM absorption (left column, Figure 12) as an increase in CDOM from major river outflows such as the Amazon and Congo following peak runoff [69], and in the upwelling region of the Arabian Sea induced by the southwesterly monsoon. In the case of the Amazon River, the CDOM in the distal plume can be seen well into the following season as it drifts slowly eastward across the Atlantic from the retroflection of the North Brazil Current [70], indicating that satellite retrievals of CDOM using the MLR can successfully track surface DOM as it evolves over time scales of weeks to years and over very long distances. The results shown in Figure 12 are broadly similar to those described in [13] for CDM at 440 nm retrieved using the GSM algorithm [71], and to the empirical algorithm of Shanmugam [31] for *a_g_*(350) (their Figure 12), although we show generally higher absorption across the equatorial regions and some parts of the Southern Ocean.

**Figure 13.**
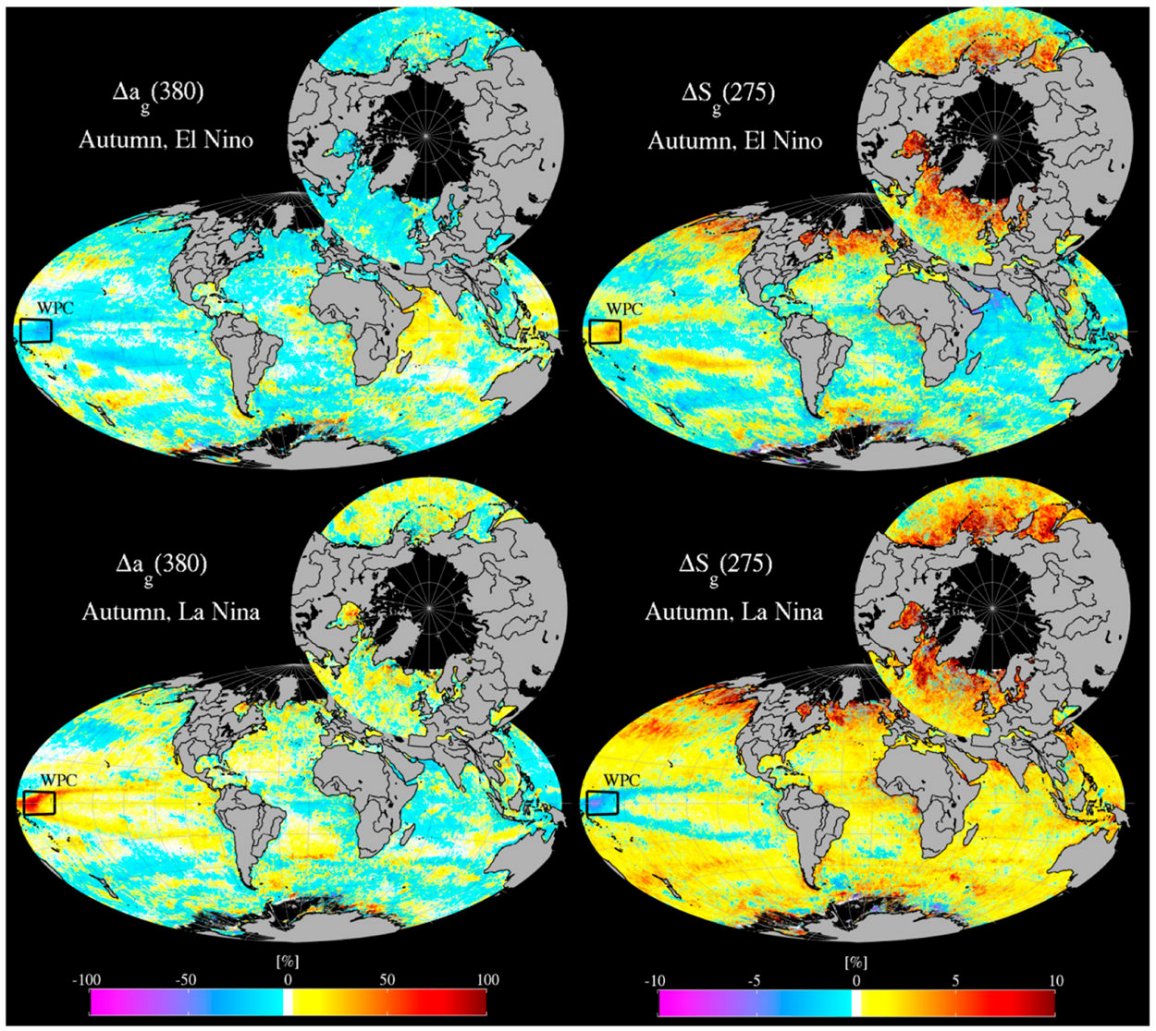
CDOM anomaly (left) and slope anomaly (right) from MLR applied to MODIS Aqua during Autumn in El Niño years (2002–2005; top panel) and La Niña years (2007, 2008, 2010, 2011; bottom panel). The Western Pacific Crescent (WPC) feature is defined here as the broad region exhibiting a notable decline in CDOM during El Niño years, and enhancement during La Niña. UV slope shows the opposite pattern, with lower slopes during La Niña, although the percentage change is roughly an order of magnitude lower. The box shows the portion of the WPC subsampled for comparison with MEI (See Figure 14).

**Figure 14.**
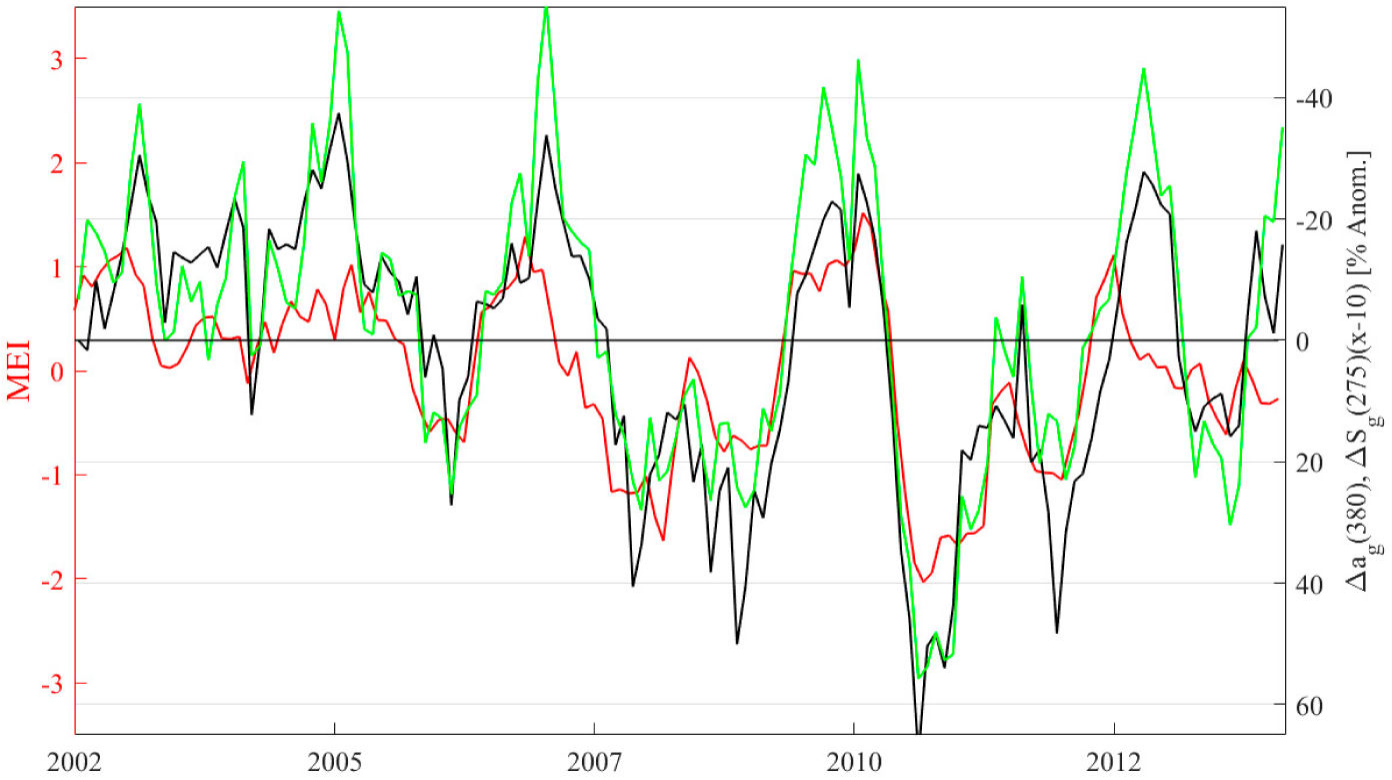
Multivariate ENSO Index (MEI, in red), CDOM anomaly at 380 nm (black), and UVB slope anomaly (green, scaled by a factor of −10 for clarity) over the entire MODIS Aqua era for the region of interest highlighted in Figure 13. Strong negative and positive correlations exist between MEI and CDOM and slope anomalies, respectively (see text).

Longer time-scale variability in the Aqua-retrieved CDOM was also apparent from the roughly twelve years of monthly, 4 km satellite composites. An examination of the monthly CDOM anomaly (Δ*a_g_*(*λ*); the monthly *a_g_*(*λ*) divided by the Aqua-era averages for each month) and slope anomaly, Δ*S_g_*(*λ*), revealed several regions characterized by sharp declines in CDOM during certain years, and elevations in others, as well as the expected inverse proportionality between CDOM and slope in the UV. Figure 13 shows an example of this from seasonal Aqua composites of Δ*a_g_*(380) and Δ*S_g_*(275) averaged over periods of El Niño (2002–2005) when surface temperatures are higher, inhibiting vertical nutrient transport and leading to lower primary productivity, and periods of La Niña (2007, 2008, 2010, 2011), which exhibit roughly the opposite dynamics. A feature in the western equatorial Pacific stands out starkly as a crescent stretching from about 10° N to 15° S and spanning nearly the entire 100° longitude range from South America to the Solomon Islands. For brevity, we refer to this as the Western Pacific Crescent (WPC). To test the link between El Niño Southern Oscillation dynamics and the CDOM anomaly in the WPC, an average of monthly Δ*a_g_*(380) within the WPC is compared with the multivariate ENSO index (MEI [72]) and Δ*S_g_*(275–295) (Figure 14). The MEI provides a convenient index for tracking the dominant characteristics associated with ENSO, namely sea-surface pressure, temperature, wind stress, and cloudiness. Positive MEI represent the warmer El Niño cycle associated with lower wind stress, a flattening in the trans-Pacific thermocline, and inhibited productivity in the equatorial Pacific, and negative MEI represents La Niña, which is cooler, and more productive. The coupling between MEI and Δ_a_g__(380) and MEI and Δ_S_g__(275) is remarkably strong and well correlated (Figure 14; r = −0.77, r = 0.80, respectively, and *p* << 0.01 in each case). As expected, CDOM and UVB slope are also very well correlated (r = −0.90, *p* << 0.01). The tight correlation between CDOM anomaly, CDOM slope anomaly and MEI may help to predict broad changes in surface CDOM in a future in which warmer sea surface temperatures are expected, particularly in the western Pacific, as the long-term warming trend leads to oceanic conditions favorable to El Niño-like conditions [73]. In fact, sustained deficits in surface CDOM available for photooxidation and microbial remineralization across the WPC, as demonstrated here, is likely to result in a lower partial pressure of CO_2_ derived from CDOM, and may therefore increase the flux of CO_2_ into the ocean from the atmosphere, although this effect would largely be offset by the decrease in solubility associated with warmer temperatures in the surface ocean. Another consequence of lower CDOM across this region in a warming regime may be decreased surface heating through CDOM absorption, potentially providing some degree of negative feedback to the surface warming trend.

## 4. Summary

The importance of characterizing and tracking change in global oceanic dissolved carbon over climatic evolutions is only possible synoptically using earth-observing technology. As methods to measure DOM sources and sinks continue to improve using laboratory and in situ optical techniques, algorithms and orbital sensor technology must keep pace. With technological and methodological improvements, however, come inevitable challenges. The nearly three decades of field data presented here are by necessity compromised in that, for example, no one standard method was employed for the measurement of spectral CDOM absorption. Similarly there is obviously no standard algorithm for global ocean color retrievals of CDOM, as the algorithms must also continuously evolve with our knowledge of the parameters they retrieve. Many approaches have proven robust in retrieving CDOM absorption and its spectral slope over the years, though most are regionally optimized with little or no provision for what ties them together (e.g., proxies for optical water types). Global algorithms have been hampered by relatively small datasets of coincident radiometry with CDOM and CDOM slope extending into the UV, and DOC retrievals have been especially challenging due to the highly variable and often unpredictable fraction of chromophoric content.

In this study we aggregate a global dataset approximately forty times the size of previous global, bio-optical databases. Naturally despite our best efforts to ensure consistency in the data through quality control, the data within are subject to error and uncertainty largely because methodologies and technology have evolved over thirty years. Quantification of the uncertainty in field estimates of the parameters retrieved here must necessarily precede uncertainty estimates in the algorithms used to derive them. Efforts are currently underway at NASA and elsewhere to do just that. New field data are always being collected and archived all over the world by various academic and public-sector agencies, but only a fraction is broadly distributed through invaluable archives like SeaBASS, in part because submission is only required of those principal investigators funded by NASA. Future algorithm development efforts should facilitate more cooperation and collaboration with other agencies collecting field data around the world encouraging sharing of data within a reasonable time after collection. To date, GOCAD and SeaBASS coverage in regions such as the Mediterranean and the oceans around Australia is astonishingly poor. Efforts must be sustained to continue bringing newly collected and historical data into GOCAD, NOMAD, and similar global, long-term bio-optical databases, and to expand them to include an even more comprehensive suite of inherent optical properties, which help support the development of more robust semi-analytical approaches. GOCAD was designed using nested and comprehensive Matlab structures conducive to expansion for both newly collected datasets as well as more complete suites of physical, optical, radiometric, and biogeochemical data. Based on our experience with SeaBASS, more rigorous quality control and documentation standards should be applied not only to recent and new submissions, but retroactively to historic data as well. While some algorithms performed better than others in this non-exhaustive comparison, it is important that algorithms continue to evolve as the data used to develop them improves, incorporating more than minor adjustments to empirical coefficients, and moving algorithms for oceanic carbon closer to theoretical, analytically-based approaches.

A representational suite of approaches, including empirical, semi-analytical, and machine-learning algorithms was evaluated against GOCAD field data for retrieving *a_g_* in six wavebands between 275 and about 490 nm, *S_g_* in eight wavebands in the UV and VIS, and DOC using a wide variety of metrics. Ultimately, the most versatile and best performing of those tested was a simple, empirical set of relationships based on multiple linear regression between four wavebands of remote sensing reflectance (440–555 nm), with the exception of DOC which also required sea surface salinity (e.g., from Aquarius) to act as a proxy to optical water type. Results varied, with CDOM retrievals showing regression coefficients to field data (r^2^) generally over 0.80 for field radiometry to within 16%−34%, depending on the wavelength, and within 33%−54% for MODIS Aqua validation. CDOM slopes retrievals were best in the UVB (e.g., r^2^ = 0.62, *MAPD* = 11% in satellite validation of *S_g_*(275–295)), while DOC algorithms only optimized well after the inclusion of salinity (r^2^ = 0.91, *MAPD* = 15%), and did not perform well in validation (e.g., RMSE = 27–29 μmol L^μ1^). Our analysis of the sensitivity of the DOC algorithm performance to factors such as salinity, DOC, water column depth, and geographic location ultimately proved inconclusive, exposing only a small anomaly involving overestimates of DOC in the southern oceans using MODIS Aqua imagery. Further validation—particularly in mid–ocean gyres where DOC varies very weakly or not at all with CDOM absorption, and salinity changes are very small—is clearly warranted prior to application of the DOC algorithm in those regions.

Application of CDOM algorithms to monthly and climatological Aqua imagery demonstrated that global retrievals of CDOM do not covary well with similar empirical retrievals of Chl, but rather appear to follow Chl on a seasonal lag depending on the region and source of dissolved material. This helps explain the lack of correlation between CDOM and Chl found in global GOCAD field data and described in previous studies, and further challenges the use of Case 1 assumptions in bio-optical remote sensing. Surface CDOM concentration varies in regions such the western equatorial Pacific by about 150% over the course of long-term climatological shifts associated with ENSO, fluctuating in tight correlation with the MEI and CDOM slope. Algorithms developed here may be applied to tracking ENSO behavior in the future, as well as observing changes in CDOM character and concentration associated with global warming.

## Figures and Tables

**Table 1.** Definition of terms, units, and abbreviations.

	Units	Definition
*a_g_*(*λ*)	m^−1^	CDOM absorption coefficient
*a_d_*(*λ*)	m^−1^	NAP absorption coefficient
*a_dg_*(*λ*)	m^−1^	NAP and CDOM absorption coefficient
*a_p_*(*λ*)	m^−1^	Particulate absorption coefficient
*b_bp_*(*λ*)	m^−1^	Particle backscattering coefficient
*b_bt_*(*λ*)	m^−1^	Total backscattering coefficient
CDOM		Colored Dissolved Organic Matter
Chl	mg m^−3^	Chlorophyll concentration
DOC, DOM	μmol L^−1^	Dissolved Organic Carbon, -Material
*E_s_*(*λ*)	W m^−2^ nm^−1^	Downwelling surface irradiance
*L_w_*(*λ*)	W m^− 2^ nm^−1^_sr_^−1^	Water leaving radiance
*L_wn_*(*λ*)	W m^− 2^ nm^−1^ sr^−1^	Normalized water leaving radiance
POC	μmol L^−1^	Particulate Organic Carbon
*R_rs_*(*λ*)	sr^−1^	Remote sensing reflectance
S_g_(*λ*_1_-*λ*_2_)	nm^−1^	Exponential slope of CDOM and in select spectral range
SPM	mg m^−3^	Suspended Particulate Material
TOC	μmol L^−1^	Total Organic Carbon
AOP		Apparent Optical Properties
GIOP		Generalized IOP Algorithm
GOCAD		Global Ocean Carbon Algorithm Database
HMW		High Molecular Weight
IOCCG		International Ocean-Colour Coordinating Group
IOP		Inherent Optical Properties
LMW		Low Molecular Weight
MLR		Multiple Linear Regression Algorithm
MODIS		Moderate Resolution Imaging Spectroradiometer
NAP		Non-Algal Particulate
NOMAD		NASA bio-Optical Algorithm Dataset
QAA		Quasi-Analytical Algorithm
RFTB		Random Forest Tree Bagger Algorithm
SAA		Semi-Analytical Algorithm
SeaBASS		SeaWiFS Bio-optical Archive and Storage System
SeaWiFS		Sea-viewing Wide Field-of-view Sensor
UV, UVA, UVB		Ultraviolet spectrum, 315–400 nm, 280–315 nm
VIS		Visible spectrum

**Table 2.** Summary of field data collection campaigns.

Numbers of Stations													
Experiment	Principal Investigators	Cruises	CDOM	CDOM & IS*	CDOM & SAT**	DOC	DOC & IS*	DOC & SAT**	Min. Lat	Max. Lat	Min. Lon	Max. Lon	Year(s)
MURI	A. Neeley, S. Freeman, J. Chaves, C. McClain	1	0	0	0	6	0	0	19.126	20.692	−157.36	−156.32	2012
EGEE3	A. Subramaniam	1	9	0	0	0	0	0	−6.003	3.327	−10.008	7.992	2006
EGEE5	A. Subramaniam	1	2	0	0	0	0	0	−5.977	−5.62	5.85	7.997	2007
MANTRA PIRANA	A. Subramaniam	5	41	2	2	0	0	0	3.386	25.002	−158.02	−42.276	2001–2003
MASS BAY	A. Subramaniam	7	39	0	13	0	0	0	41.85	42.619	−70.895	−70.228	2002–2005
IOFFE	A. Khrapko, S. Ershova	1	164	56	51	0	0	0	−66.46	48.59	−67.98	−5.54	2001–2002
B01	A. Mannino	2	15	5	0	16	5	0	36.713	37.786	−76.018	−74.644	2005
B02	A. Mannino	2	28	18	6	30	17	6	36.685	38.918	−76.069	−74.502	2005
B03	A. Mannino	2	26	0	14	29	0	16	36.413	38.87	−76.022	−74.499	2006
B04	A. Mannino	2	30	19	6	46	19	9	36.502	38.908	−76.019	−74.299	2006
B05	A. Mannino	1	15	0	5	15	0	5	36.431	38.586	−76.017	−73.517	2006
BIOD01	A. Mannino	1	15	11	1	15	11	1	42.361	43.574	−70.696	−69.863	2007
BIOD02	A. Mannino	1	17	14	6	17	14	6	42.593	43.708	−70.78	−69.691	2007
BIOD03	A. Mannino	1	13	12	0	12	11	0	41.201	42.812	−70.771	−70.445	2007
CBM01	A. Mannino	1	2	0	0	0	0	0	36.965	37.17	−76.172	−76.029	2004
CBM02	A. Mannino	1	4	0	3	0	0	0	36.987	37.182	−76.163	−76.018	2004
CBM03	A. Mannino	1	4	0	2	0	0	0	36.987	37.182	−76.163	−76.018	2004
CBM04	A. Mannino	1	4	0	0	0	0	0	36.987	37.182	−76.163	−76.018	2004
CBM05	A. Mannino	1	4	0	3	0	0	0	36.987	37.182	−76.163	−76.018	2004
CBM06	A. Mannino	1	4	0	3	0	0	0	36.987	37.182	−76.163	−76.018	2005
CBM07	A. Mannino	1	3	0	2	0	0	0	37.046	37.182	−76.138	−76.018	2005
CBM08	A. Mannino	1	3	0	0	0	0	0	37.047	37.182	−76.137	−76.019	2005
CBM09	A. Mannino	1	5	0	0	0	0	0	36.987	37.181	−76.161	−76.017	2005
CBM10	A. Mannino	1	4	0	3	0	0	0	36.987	37.181	−76.161	−76.017	2005
CBM11	A. Mannino	1	4	0	0	0	0	0	37.046	37.182	−76.136	−76.019	2006
CBM12	A. Mannino	1	4	0	0	0	0	0	36.987	37.182	−76.161	−76.018	2006
COI1	A. Mannino	1	4	2	3	4	2	3	36.69	36.969	−76.017	−75.713	2007
COI2	A. Mannino	1	4	1	3	4	1	3	36.69	36.969	−76.017	−75.71	2007
COI3	A. Mannino	1	4	1	0	3	0	0	36.69	36.969	−76.017	−75.713	2007
CV1	A. Mannino	1	53	0	24	51	0	23	35.745	42.498	−75.706	−65.736	2009
CV2	A. Mannino	1	69	0	35	69	0	35	36.475	43.062	−75.785	−66.088	2009
CV3	A. Mannino	1	43	0	9	43	0	9	37.089	43.112	−75.677	−65.779	2010
CV4	A. Mannino	1	79	0	30	78	0	30	36.073	44.233	−75.911	−65.772	2010
CV5	A. Mannino	1	67	0	24	63	0	24	36.142	44.299	−75.859	−65.775	2010
CV6	A. Mannino	1	92	0	18	92	0	18	36.187	43.928	−75.789	−65.768	2011
D01	A. Mannino	1	4	2	2	4	2	2	36.797	36.966	−76.02	−75.719	2005
D02	A. Mannino	1	6	6	6	6	6	6	36.805	36.973	−76.02	−75.712	2005
D03	A. Mannino	1	5	2	0	5	2	0	36.803	36.968	−76.018	−75.718	2006
D04	A. Mannino	2	6	3	0	6	3	0	36.801	36.966	−76.015	−75.64	2006
OCV1	A. Mannino	1	26	26	18	26	26	18	40.218	40.731	−74.153	−73.479	2007
OCV2	A. Mannino	1	22	18	2	22	18	2	40.208	40.724	−74.151	−73.451	2007–2009
OCV3	A. Mannino	1	8	8	0	8	8	0	40.392	40.739	−74.156	−73.554	2008
OCV5	A. Mannino	1	11	11	3	11	11	3	39.584	41.028	−73.901	−71.749	2009
PL6	A. Mannino	1	4	0	3	4	0	3	36.802	36.968	−76.017	−75.713	2007
GOMECC-2	A. Mannino, J. Salisbury	1	67	0	1	92	0	2	25.999	43.032	−90.809	−68.01	2012
GEO-CAPE	A. Mannino, M. Mulholland	1	59	53	6	54	51	6	38.098	39.17	−76.491	−76.084	2011
MONTEREY BAY	B. Arnone, R. Gould	1	57	51	11	0	0	0	36.271	36.988	−123.13	−121.81	2003
WOCE P14S P15S	B. Tilbrook	1	0	0	0	107	0	0	−66.99	−0.003	−174.79	173.982	1996
Carbon Transport MS R.	C. Del Castillo	2	9	0	6	0	0	0	28.295	28.925	−89.743	−89.411	2001–2003
GasEx	C. Del Castillo	1	44	10	3	0	0	0	−53.75	−50.14	−38.554	−36.622	2008
Big Bend	C. Hu	10	156	61	36	0	0	0	29.168	29.67	−83.635	−83.201	2010–2011
GEO-CAPE CBODAQ	C. Hu	2	16	12	0	23	11	2	38.098	39.17	−76.487	−76.083	2011
GOM Oil Spill	C. Hu	3	10	0	1	0	0	0	28.584	29.1	−88.42	−87.323	2010
Glider calibration	C. Hu	1	8	0	4	0	0	0	27.452	28.465	−83.992	−83.072	2009–2011
Glider validation	C. Hu	1	13	0	6	0	0	0	27.356	27.48	−83.115	−83.051	2009
SWFL	C. Hu	3	21	3	2	0	0	0	24.827	26.49	−82.318	−81.141	2010–2011
Tampa Bay	C. Hu	5	85	71	5	0	0	0	27.578	27.991	−82.783	−82.408	2008–2012
West Florida Shelf	C. Hu	5	134	67	53	0	0	0	25.057	28.439	−83.785	−81.143	2005–2008
MOCE	C. Trees, D. Clark	2	40	0	17	0	0	0	21.793	36.875	−122	−105.75	1992–1999
BowdoinBuoy	C. Roesler	1	2	0	0	0	0	0	43.762	43.795	−69.988	−69.947	2011
PenBaySurvey	C. Roesler	1	0	0	0	0	0	0	44.26	44.26	−68.983	−68.983	2008
San Diego Coastal Project	D. Stramski, M. Stramska	1	15	0	0	0	0	0	32.558	32.758	−117.26	−117.13	2004–2006
NASA Gulf of Maine	D. Phinney, D. Phinney, J. Brown	4	46	0	6	0	0	0	41	44.245	−70.567	−67.162	1998–1999
NOAA Gulf of Maine	D. Phinney, D. Phinney, J. Brown	4	37	0	4	0	0	0	40.209	44.344	−70.056	−65.549	1996–1998
Panama City Florida	D. Phinney, D. Phinney, J. Brown	1	0	0	0	0	0	0	30.167	30.172	−85.857	−85.852	2001
Plumes and Blooms	D. Siegel	5	88	1	22	0	0	0	34.024	34.464	−120.56	−119.28	2001–2003
CLIVAR A13.5 2010	D. Hansell	1	0	0	0	64	0	4	−54	4.62	−3.002	1.835	2010
CLIVAR I05 2009	D. Hansell	1	0	0	0	1	0	0	−31.19	−31.19	82.564	82.564	2009
CLIVAR I08S 2007	D. Hansell	1	0	0	0	28	0	2	−65.71	−28.32	81.962	95.014	2007
CLIVAR P02 2004	D. Hansell	1	0	0	0	56	0	7	29.991	32.644	−177.99	179.545	2004
CLIVAR P16N 2006	D. Hansell	1	0	0	0	78	0	2	−17	56.28	−153.22	−150	2006
CLIVAR P16S 2005	D. Hansell	1	0	0	0	57	0	1	−71	−16	−150.04	−149.91	2005
CLIVAR P18 2007	D. Hansell	1	0	0	0	72	0	4	−68.91	22.7	−110.04	−102.54	2007
HLY-02-01	D. Hansell	1	0	0	0	21	0	0	64.98	73.431	−169.14	−154.4	2002
HLY-02-03	D. Hansell	1	0	0	0	38	0	1	65.668	73.698	−168.86	−151.94	2002
HLY-0403	D. Hansell	1	0	0	0	36	0	0	65.661	73.827	−168.9	−152.02	2004
SR03	D. Hansell	1	0	0	0	24	0	0	−65.57	−44.38	139.658	146.189	2008
WOCE AR01 A05	D. Hansell	1	0	0	0	45	0	6	24.499	27.622	−79.937	−14.224	1998
ACE-ASIA	G. Mitchell, M. Kahru	1	45	22	10	0	0	0	28.207	38.905	−177	178.05	2001
AMLR	G. Mitchell, M. Kahru	7	47	0	7	0	0	0	−63.01	−57.5	−68.186	−53.296	2000–2007
Aerosols Index	G. Mitchell, M. Kahru	1	21	3	9	0	0	0	−34.53	27.368	−60.615	85.166	1999
CALCOFI	G. Mitchell, M. Kahru	16	179	8	29	0	0	0	29.847	36.057	−124.33	−117.3	1996–2002
Sea_of_Japan	G. Mitchell, M. Kahru	1	17	1	1	0	0	0	34.503	43.302	128.883	139.883	1999
Arc00	G. Cota	1	14	0	0	0	0	0	70.328	72.412	−167.59	−144.63	2000
LAB97	G. Cota	1	10	0	0	0	0	0	44.137	60.38	−58.19	−43.999	1997
Lab2000	G. Cota	1	6	0	2	0	0	0	49.518	60.047	−58.749	−48.899	2000
Lab96	G. Cota	1	10	0	0	0	0	0	52.08	60.999	−58.006	−47.908	1996
ORCA Ches. Light	G. Cota	1	3	0	2	0	0	0	36.9	36.9	−75.71	−75.71	2000
Res95	G. Cota	1	1	0	0	0	0	0	74.645	74.645	−95.91	−95.91	1995
Res96	G. Cota	1	6	0	0	0	0	0	74.644	74.646	−94.915	−94.905	1996
NSF-BWZ	G. Mitchell	2	0	0	0	0	0	0	−62.26	−60.63	−58.375	−55.6	2004–2006
Benthic Ecol. from	H. Dierssen, R. Zimmerman	4	8	0	0	0	0	0	24.723	29.847	−85.382	−80.705	2005–2006
Kieber Photochemistry 03	H. Sosik	1	17	0	5	0	0	0	35.278	41.075	−75.218	−71.127	2003
MVCO	H. Sosik	34	129	0	23	0	0	0	41.143	41.342	−70.638	−70.415	2005–2011
GOCAL	J.R.V Zaneveld, W.S. Pegau	6	140	0	48	0	0	0	22.914	31.116	−114.64	−107.75	1996–1999
PREPP	J. Chen	5	47	0	0	0	0	0	22.15	22.555	113.673	114.43	2001
SAB Mapping	J. Nelson, A. Subramaniam	2	18	0	10	0	0	0	30.823	31.993	−81.024	−80.221	2005
GEOTRACES	J. Chaves	1	0	0	0	9	0	0	17.35	36.766	−24.496	−12.825	2010
COOA	J. Salisbury, D. Vandemark, C. Hunt	3	0	0	0	14	0	4	42.861	43.757	−70.66	−69.782	2008
NOAA CSC	J. Brock, A. Subramanian, K. Waters	1	10	0	0	0	0	0	31.335	31.965	−81.128	−80.454	1996
BOA	K. Carder	1	62	62	0	0	0	0	27.579	59.841	−91.768	−15.49	1991–1993
EcoHAB	K. Carder	19	398	208	126	0	0	0	25.3	27.572	−84.394	−81.259	1999–2003
Okeechobee	K. Carder	1	4	4	0	0	0	0	27.149	27.199	−80.794	−80.788	1997
Redtide	K. Carder	2	13	11	7	0	0	0	27.289	28.098	−83.253	−82.866	2005
TOTO	K. Carder	3	86	75	40	0	0	0	24.884	27.5	−82.776	−77.587	1998–2000
ACE-INC	L.W. Harding_Jr.,M. Mallonee, A. Magnuson	6	21	0	0	0	0	0	38.303	38.754	−76.62	−76	2002–2003
BIOCOMPLEXITY	L.W. Harding_Jr.,M. Mallonee, A. Magnuson	11	55	0	15	0	0	0	36.863	39.349	−76.451	−75.878	2001–2004
LMER-TIES	L.W. Harding_Jr.,M. Mallonee, A. Magnuson	17	220	0	22	0	0	0	36.866	39.421	−76.517	−75.749	1996–2000
SGER	L.W. Harding_Jr.,M. Mallonee, A. Magnuson	1	11	0	5	0	0	0	36.95	38.5	−76.481	−75.998	2003
ONR-MAB	L.W. Harding_Jr.,M. Mallonee, A. Magnuson M.S. Twardowski, A.H.	2	31	0	0	0	0	0	36.4	39.134	−75.949	−71.993	1996–1997
Ocean Color Cal Val	Barnard, J.R.V. Zaneveld	1	14	13	0	0	0	0	40.208	40.511	−74.054	−73.448	2007
Tokyo Bay	M. Kishino	1	1	0	0	0	0	0	35.223	35.223	139.718	139.718	1984
Global CDOM	N. Nelson, D. Siegel	2	19	0	3	0	0	0	−8.458	7.004	−140.07	−124.35	2005–2006
CLIVAR	N. Nelson, D. Siegel, C. Carlson	9	80	17	4	45	8	4	−68.36	59.5	−150	95.028	2003–2008
TAO 2005	N. Nelson, D. Siegel, C. Carlson	1	61	0	9	0	0	0	−8.89	12	−140.2	−124.35	2005
TAO 2006	N. Nelson, D. Siegel, C. Carlson	2	78	0	7	0	0	0	−8.458	10.012	−140.17	−123.55	2006
BBOP	N. Nelson, D. Siegel	116	78	7	9	40	0	6	31.446	31.815	−64.991	−64.019	1994–2011
Active Fluorescence 2001	R. Morrison, H. Sosik	1	4	0	0	0	0	0	31.919	40.097	−70.528	−69.784	2001
Kieber Photochemistry 02	R. Morrison, H. Sosik	1	69	0	7	0	0	0	38.709	42.511	−75.564	−67.599	2002
GLOBEC	R. Morrison, H. Sosik	5	23	0	9	0	0	0	41.753	43.799	−70.445	−65.685	1997–1999
FRONT	R. Morrison, H. Sosik	3	9	0	4	0	0	0	42.245	40.985	−70.558	−71.75	2000–2002
CLIVAR A16N 2003	R. Freely	1	0	0	0	69	0	8	−6.004	63.295	−29.001	−19.994	2003
CLIVAR A16S 2005	R. Freely	1	0	0	0	49	0	3	−60.01	−2.334	−36.21	−24.997	2005
CLIVAR A20 2003	R. Freely	1	0	0	0	27	0	3	7.064	42.637	−53.51	−51.12	2003
CLIVAR A22 2003	R. Freely	1	0	0	0	37	0	6	11.001	39.857	−69.932	−64.161	2003
CLIVAR I09N 2007	R. Freely	1	0	0	0	51	0	2	−28.31	18.004	86.782	95.013	2007
North Carolina 2005	R. Stumpf, P. Tester	5	32	4	20	0	0	0	34.096	35.433	−76.693	−75.756	2005
North Carolina 2006	R. Stumpf, P. Tester	2	12	0	6	0	0	0	34.014	35.228	−76.388	−76.028	2006
Chesapeake Light Tower	R. Zimmerman, G. Cota	3	71	59	32	0	0	0	36.803	36.969	−76.101	−75.551	2005–2007
North Sea	R. Doerffer	1	32	0	0	0	0	0	52.226	55.367	0.591	8.123	1994
ICESCAPE	S.B. Hooker, A. Neeley	3	1609	31	31	85	28	6	56.211	73.828	−168.98	−150.44	2001–2011
B07	S.B. Hooker, M.E. Russ	1	11	0	3	20	0	0	42.65	43.18	−70.868	−70.616	2009
MALINA	S.B. Hooker, V. Wright	1	25	22	0	28	1	0	69.246	72.054	−140.83	−126.5	2009
B08	S.B. Hooker, J. Chaves	1	0	0	0	2	0	1	31.667	31.698	−64.169	−64.164	2009
COASTAL	S.B. Hooker, M.E. Russ	1	13	0	10	0	0	0	42.708	43.434	−70.794	−69.865	2008
USM pCO_2_	S. Lohrenz	1	1	0	0	0	0	0	28.858	28.858	−89.47	−89.47	2005
Catlin Arctic Survey	V. Hill	1	8	0	0	0	0	0	78.771	78.771	−104.72	−104.72	2011
AMT	W. Balch	5	23211	7703	1918	0	0	0	−47.27	49.716	−55.455	18.611	2005–2011
Gulf of Maine	W. Balch	33	8077	0	3251	0	0	0	42.683	44.058	−70.267	−66.172	2005–2008
Scotia Prince Ferry	W. Balch	47	11521	51	8397	0	0	0	43.604	43.798	−70.026	−66.164	2001–2004
2009oct Chesapeake	W.J. Rhea	1	13	10	4	0	0	0	38.136	39.062	−76.448	−76.229	2009
Totals		535	48574	8857	14568	1957	255	302	−71	78.771	−177.99	179.545	1984–2012

* IS is in situ *R_rs_*(*λ*),

** SAT is satellite *R_rs_*(*λ*)

**Table 3.** Coefficients of the MLR algorithm for retrieving CDOM absorption (*ag*(*λ*)) following Equation (8) and metrics of fit for the optimization data set.

	ß_0_	ß_1_	ß_2_	ß_3_	ß_4_	N	r^2′^	*RMSD* [m^−1^]	*MAPD* [%]	*%Bias* [%]	Threshold [m^−1^]
**MODIS**											
[nm]		443	488	531	547						

275	0.089	−0.540	−1.142	3.444	−1.875	100	0.56	0.499	16	−1.4	4.825
355	−2.246	−1.186	−0.558	2.912	−1.336	116	0.90	0.102	16	−0.9	0.9104
380	−2.263	−0.300	−1.882	3.831	−1.787	86	0.83	0.040	15	−1.9	0.4341
412	−2.535	−0.563	−1.294	1.606	0.170	483	0.87	0.048	28	−4.3	0.36419
443	−3.287	−0.727	−0.922	1.278	0.261	462	0.85	0.026	29	−4.3	0.1984
488	−3.722	−0.377	−1.429	1.424	0.300	490	0.82	0.016	34	−5.9	0.1114

**SeaWiFS**											
[nm]		443	490	510	555						

275	−2.477	−2.880	2.225	0.480	−0.252	174	0.76	0.659	25	−2.2	4.825
355	−4.199	−2.563	1.214	0.955	−0.040	189	0.87	0.118	26	−2.2	0.9104
380	−4.544	−1.808	0.175	1.181	0.001	150	0.80	0.055	26	−2.4	0.4341
412	−6.004	−0.861	−0.006	−0.346	0.515	8066	0.37	0.035	56	−13.0	0.36419
443	−6.410	−0.743	−0.145	−0.367	0.547	8037	0.33	0.026	58	−13.6	0.1984
490	−7.014	−0.736	0.142	−0.796	0.678	7978	0.28	0.016	65	−15.5	0.1114

**Table 4.** Coefficients of the MLR algorithm for retrieving CDOM spectral slope (*S_g_*(*λ*)) following Equation (8) and metrics of fit for the optimization data set.

*λ*	ß_0_	ß_1_	ß_2_	ß_3_	ß_4_	N	r^2′^	*RMSD* [nm^−1^]	*MAPD* [%]	*%Bias* [%]
**MODIS**										
[nm]		443	488	531	547					

275–295	−3.289	0.270	−0.335	1.051	−0.921	322	0.61	0.002	6.4	−0.3
290–600	−3.471	0.127	−0.251	1.025	−0.843	322	0.38	0.002	6.2	−0.3
300–600	−3.607	0.044	−0.153	0.881	−0.722	324	0.30	0.001	5.7	−0.3
350–400	−3.924	−0.242	0.055	0.935	−0.710	331	0.26	0.001	6.8	−0.3
350–600	−3.908	−0.204	0.098	0.609	−0.463	331	0.22	0.001	6.3	−0.3
380–600	−3.912	−0.152	0.127	0.236	−0.173	340	0.14	0.001	6.3	−0.3
412–600	−4.219	−0.180	0.137	0.168	−0.131	782	0.16	0.002	7.4	−0.5
412–555	4.195	−0.162	0.147	0.096	−0.084	760	0.10	0.002	7.6	−0.5

**SeaWiFS**										
[nm]		443	490	510	555					

275–295	−3.012	0.427	−0.459	0.357	−0.228	424	0.77	0.002	6.8	−0.4
290–600	−3.425	0.131	−0.085	0.145	−0.130	424	0.46	0.002	6.6	−0.4
300–600	−3.615	0.004	0.014	0.160	−0.129	426	0.29	0.002	6.0	−0.3
350–400	−3.968	−0.298	0.178	0.301	−0.150	433	0.23	0.002	7.4	−0.4
350–600	−4.058	−0.288	0.091	0.356	−0.138	433	0.33	0.001	6.9	−0.4
380–600	−4.072	−0.226	0.088	0.208	−0.051	445	0.32	0.002	7.2	−0.3
412–600	−4.498	−0.466	0.690	−0.202	−0.015	8550	0.06	0.004	28.5	−5.2
412–555	−4.533	−0.455	0.683	−0.214	−0.012	8425	0.05	0.004	28.2	−5.1

**Table 5.** Coefficients of the MLR algorithm for retrieving DOC following Equation (8) and metrics of fitfor the optimization data set.

Algorithm	ß_0_	ß_1_	ß_2_	ß_3_	ß_4_	N	r^2′^	*RMSD* [μmol L^−1^]	*MAPD* [%]	*%Bias* [%]
**MODIS**										
[nm]		443	488	531	547					
MLR1	4.923	0.641	−2.424	3.503	1.692	183	0.76	23.9	13.9	−1.5
**SeaWiFS**										
[nm]		443	490	510	555					
MLR1	5.272	0.526	−2.982	2.623	0.089	246	0.68	30.3	18.8	−3.0
***a_g_*(355) and Salinity**										
		*a_g_*(355)	*Sal*							
MLR2	192.718	26.790	−3.558	-	-	464	0.91	15.2	10.6	0.0

**Table 6.** Validation of algorithms for retrieving CDOM absorption (ag(*λ*)).

Algorithm	*γ*	N	r^2^	*RMSE*	*MAPD*	*%Bias*

	[nm]			[m^−1^]	[%]	[%]
**MODIS-Aqua**						
**MLR**	**275**	**186**	**0.47**	**2.499**	**33**	**−17**
**MLR**	**380**	**188**	**0.45**	**0.117**	**54**	**−1**
**MLR**	**412**	**7626**	**0.33**	**0.068**	**33**	**−10**
RFTB	275	191	0.50	3.100	78	37
RFTB	380	243	0.47	0.400	102	28
RFTB	412	6820	0.30	0.100	52	18
Shan11	350	237	0.45	1.669	134	108
Shan11	412	7748	0.28	0.287	92	72
TS11	412	7299	0.39	0.201	102	86
GIOP	412	6116	0.30	0.077	40	−12
QAA	412	6133	0.14	0.137	59	18

**MODIS-Terra**						
**MLR**	**275**	**291**	**0.43**	**2.746**	**48**	**−26**
**MLR**	**380**	**326**	**0.32**	**0.337**	**45**	**−29**
**MLR**	**412**	**10612**	**0.19**	**0.081**	**35**	**−1**
RFTB	275	171	0.35	2.950	72	22
RFTB	380	269	0.35	0.380	125	21
RFTB	412	6962	0.20	0.110	63	34
Shan11	350	345	0.35	1.308	97	75
Shan11	412	10734	0.16	0.311	129	108
TS11	412	9607	0.20	0.202	109	87
GIOP	412	7976	0.12	0.108	51	11
QAA	412	8048	0.05	0.200	91	52

**SeaWiFS**						
**MLR**	**275**	**342**	**0.25**	**2.976**	**49**	**−57**
**MLR**	**380**	**418**	**0.32**	**0.318**	**58**	**−53**
**MLR**	**412**	**10233**	**0.10**	**0.081**	**47**	**23**
RFTB	275	199	0.38	2.660	90	40
RFTB	380	423	0.36	0.370	209	41
RFTB	412	11594	0.06	0.110	55	27
Shan11	350	444	0.51	1.425	108	86
Shan11	412	10451	0.26	0.229	100	79
TS11	412	8890	0.20	0.250	152	128
GIOP	412	7863	0.11	0.085	45	11
QAA	412	7904	0.07	0.162	80	53

**Table 7.** Validation of algorithms for retrieving CDOM spectral slope (*S_g_*).

Algorithm	Waveband	N	r^2^	*RMSE*	*MAPD*	*%Bias*

	[nm]			[nm^−1^]	[%]	[%]
**MODIS-Aqua**						
**MLR**	**275–295**	**187**	**0.62**	**0.0034**	**11**	**6**
**MLR**	**300–600**	**213**	**0.15**	**0.0023**	**10**	**8**
**MLR**	**412–555**	**7825**	**0.06**	**0.0040**	**32**	**23**
RFTB	275–295	214	0.58	0.0033	8	−1
RFTB	300–600	244	0.20	0.0019	8	4
Shan11	350–600	188	0.01	0.2750	427	366
TS11	412–555	6223	0.02	0.0290	217	−209

**MODIS-Terra**						
**MLR**	**275–295**	**284**	**0.41**	**0.0039**	**12**	**3**
**MLR**	**300–600**	**318**	**0.16**	**0.0024**	**10**	**9**
**MLR**	**412–555**	**10719**	**0.06**	**0.0036**	**27**	**20**
RFTB	275–295	242	0.43	0.0000	10	−3
RFTB	300–600	271	0.11	0.0000	8	5
Shan11	350–600	297	0.03	0.1250	255	176
TS11	412–555	8078	0.00	0.0280	208	−203

**SeaWiFS**						
**MLR**	**275–295**	**350**	**0.44**	**0.0047**	**14**	**6**
**MLR**	**300–600**	**417**	**0.11**	**0.0021**	**8**	**4**
**MLR**	**412–555**	**10883**	**0.03**	**0.0029**	**17**	**−12**
RFTB	275–295	350	0.37	0.0053	11	−7
RFTB	300–600	418	0.09	0.0022	9	5
Shan11	350–600	372	0.01	0.1320	202	140
TS11	412–555	4716	0.03	0.0290	203	−198

**Table 8.** Validation of algorithms for retrieving DOC.

Algorithm	N	r^2^	*RMSE*	*MAPD*	*%Bias*

			[μmol L^−1^]	[%]	[%]
**MODIS-Aqua**					
MLR1	164	0.23	40.8	41	24.7
**MLR2**	**382**	**0.89**	**27.8**	**16**	**−13.0**
RFTB	161	0.57	27.3	26	13.8

**MODIS-Terra**					
MLR1	158	0.23	40.2	32	13.9
**MLR2**	**369**	**0.90**	**26.7**	**15**	**−12.3**
RFTB	114	0.47	29.4	27	12.1

**SeaWiFS**					
MLR1	274	0.29	34.2	34	4.5
**MLR2**	**339**	**0.89**	**28.9**	**15**	**−14.2**
RFTB	182	0.30	25.3	23	6.7
